# Novel high throughput pooled shRNA screening identifies NQO1 as a potential drug target for host directed therapy for tuberculosis

**DOI:** 10.1038/srep27566

**Published:** 2016-06-14

**Authors:** Qing Li, Ahmad F. Karim, Xuedong Ding, Biswajit Das, Curtis Dobrowolski, Richard M. Gibson, Miguel E. Quiñones-Mateu, Jonathan Karn, Roxana E. Rojas

**Affiliations:** 1 Department of Medicine, Case Western Reserve University & University Hospitals, Cleveland, Ohio, United States of America; 2 Department of Molecular Biology and Microbiology, Case Western Reserve University & University Hospitals, Cleveland, Ohio, United States of America; 3 University Hospitals Translational Laboratory, University Hospitals Case Medical Center, Cleveland, Ohio, United States of America; 4 Center for AIDS Research (CFAR), Case Western Reserve University & University Hospitals, Cleveland, Ohio, United States of America; 5 Department of Pathology, Case Western Reserve University & University Hospitals, Cleveland, Ohio, United States of America

## Abstract

Chemical regulation of macrophage function is one key strategy for developing host-directed adjuvant therapies for tuberculosis (TB). A critical step to develop these therapies is the identification and characterization of specific macrophage molecules and pathways with a high potential to serve as drug targets. Using a barcoded lentivirus-based pooled short-hairpin RNA (shRNA) library combined with next generation sequencing, we identified 205 silenced host genes highly enriched in mycobacteria-resistant macrophages. Twenty-one of these “hits” belonged to the oxidoreductase functional category. NAD(P)H:quinone oxidoreductase 1 (NQO1) was the top oxidoreductase “hit”. NQO1 expression was increased after mycobacterial infection, and NQO1 knockdown increased macrophage differentiation, NF-κB activation, and the secretion of pro-inflammatory cytokines TNF-α and IL-1β in response to infection. This suggests that mycobacteria hijacks NQO1 to down-regulate pro-inflammatory and anti-bacterial functions. The competitive inhibitor of NQO1 dicoumarol synergized with rifampin to promote intracellular killing of mycobacteria. Thus, NQO1 is a new host target in mycobacterial infection that could potentially be exploited to increase antibiotic efficacy *in vivo*. Our findings also suggest that pooled shRNA libraries could be valuable tools for genome-wide screening in the search for novel druggable host targets for adjunctive TB therapies.

Despite available treatments, tuberculosis (TB) remains one of the most deadly infectious diseases^1^. One major factor fueling TB’s re-emergence is the rise of antibiotic-resistant isolates of *Mycobacterium tuberculosis*^2^,^3^. Current TB drug regimes require combinations of multiple drugs that have to be taken for six months or longer^3^,^4^,^5^. Non-compliance with this lengthy treatment and poor access to TB drugs are key contributors to the emergence of multi- and extensively drug-resistant (MDR and XDR) *M. tuberculosis* strains^3^,^6^. Efforts to develop new anti-TB drugs have focused on targeting the bacillus. However, this approach has faced numerous obstacles and the current development pipeline for antibiotics against *M. tuberculosis* is limited^2^. A more promising approach may be the development of host-directed therapeutics (HDT) to supplement antibiotic therapy and shorten the course of treatment. Successful HDT may also improve the efficacy of second line therapy in drug-resistant TB and/or decrease lung pathology to preserve function^7^.

The concept of drugs targeting host pathways to fight bacterial infection is relatively new and recent studies support the feasibility of this strategy^8^. Intracellular *M. tuberculosis* growth was controlled by inhibitors of the PBK/AKT1 network^9^ and by imatinib mesylate (STI-571, Gleevec), which inhibits ABL1, ABL2 and related tyrosine kinases^10^. Imatinib, a drug for chronic myelogenous leukemia, not only decreased *M. tuberculosis* growth *in vitro* but also reduced bacterial load and associated pathology in *M. tuberculosis* -infected mice. These findings demonstrate the potential of HDT to treat TB and provide a rationale for our efforts to obtain a precise and comprehensive definition of the host molecules involved in *M. tuberculosis* infection.

A key feature of *M. tuberculosis* pathogenesis is the ability of the bacteria to survive and replicate in host phagocytic cells^11^,^12^,^13^. Due to this central role in *M. tuberculosis* infection, macrophage function offers numerous targets for HDT. Although several crucial macrophage functions that control (autophagy, iNOS, ROS, cationic peptides) or promote (lipid bodies, iron acquisition) *M. tuberculosis* growth have been defined, many of the genes that regulate these functions remain unknown. As the first step in developing macrophage-based HDT, the identification of the full range of host molecules that participate in *M. tuberculosis*’ intracellular life cycle needs to be addressed.

One approach to identify genes/proteins that play a role in specific biological process is through functional inhibition by RNA interference (RNAi). A handful of RNAi screenings aimed at identifying host factors that control mycobacterial entry and/or survival in macrophages have been reported^14^,^15^,^16^. All these previous studies used silencing RNAs (siRNAs) in multiwell-based assays. A function-based gene identification screening using a pooled barcoded short hairpin RNA (shRNA) library and massive parallel next generation sequencing (NGS) has been recently reported for the identification of cancer drug targets^17^. This method offers improvements in speed and scale compared to plate-based screening. We have adapted this technology to the study of mycobacteria-infected macrophages, and here we report the first cell-based pooled shRNA barcode screening for identification of key host factors that regulate mycobacterial entry and adaptation to the intracellular environment.

The goal of the current studies was to identify and characterize new host molecule(s) with potential to serve as targets of HDT for TB. Using a pooled human lentiviral- shRNA sub-library (DECIPHER™), we uncovered a set of 205 host genes that promote mycobacterial entry and/or survival in macrophages. Among them, genes belonging to the oxidoreductase functional group were overrepresented. The NAD(P)H dehydrogenase, quinone 1 (NQO1), a major target of the Nrf2/ARE stress response pathway, was the top oxidoreductase “hit” and was validated with single shRNAs and chemical inhibitors in both THP1 cells and primary monocyte derived macrophages (MDM). *M. tuberculosis* infection up-regulated NQO1 expression, and NQO1-deficient macrophages exhibited increased differentiation, NF-κB activation, TNF-α and IL1β secretion and autophagy upon mycobacterial infection. This suggests that mycobacteria hijack NQO1 to down-regulate pro-inflammatory and anti-bacterial functions. The NQO1 inhibitor dicoumarol decreased mycobacterial survival in macrophages and synergized with rifampin in the intracellular killing of mycobacteria. Thus, NQO1 and other oxidoreductases could represent novel targets for HDT against TB.

Together our data suggests that barcoded shRNA libraries could be valuable tools for genome-wide screening in the quest to discover novel host drug targets for development of adjunctive TB therapies.

## Results

### High-throughput shRNA library screening for identification of host factors required for mycobacteria entry and intracellular survival

**Figure 1:**
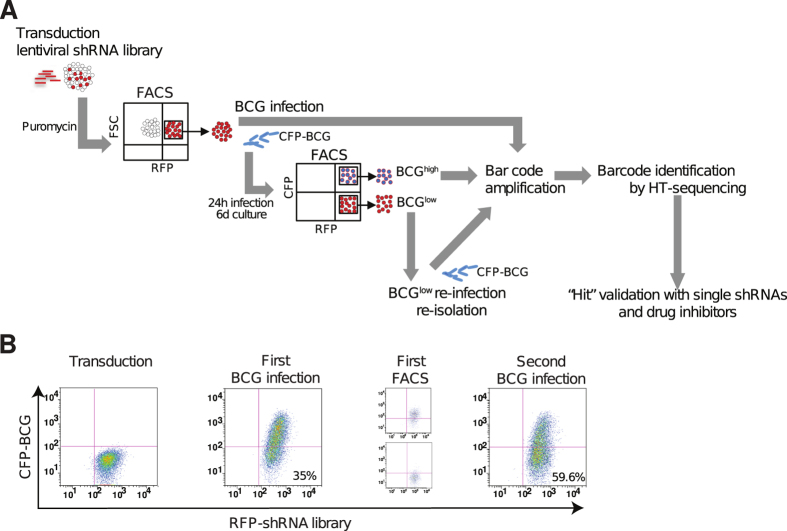
Experimental approach for screening the DECIPHER™ pooled shRNA library in mycobacteria-infected THP1 cells. (**A**) THP1 cells were transduced with lentiviral particles encoding 27,500 shRNA sequences targeting 5,043 genes (5–6 shRNAs/mRNA) and expressing red fluorescence protein (RFP). The shRNA^+^ clones were then selected (puromycin, RFP expression), expanded in culture and infected with BCG expressing cyan fluorescent protein (CFP-BCG). Six days after infection, cell clones with high bacterial loads (BCG^high^) and those that were either uninfected or harbor low number of bacteria (BCG^low^) were separated by FACS based on levels of CFP expression. After expansion in culture, BCG^low^ cells were re-infected and re-isolated to enrich the phenotype. DNA was isolated from BCG^high^-, BCG^low^- and uninfected cells, and barcodes were PCR-amplified. Barcodes sequences were identified and quantified by high-throughput Illumina sequencing (HT-sequencing). “Hits” were validated in THP1 cells and MDM with single shRNAs and small molecule inhibitors. (**B**) THP1 cells were analyzed by flow cytometry after transduction with lentiviral particles and selection with puromycin and FACS (Transduction), first round of infection with BCG (First BCG infection), purification by FACS of BCG^high^ and BCG^low^ cells after first infection (First FACS) and re-infection of BCG^low^ cells (Second BCG infection).

Novel macrophage proteins required for mycobacterial entry, intracellular survival and replication constitute attractive drug targets because their inhibition is predicted to increase the ability of the host to control *M. tuberculosis* infection. To identify these factors in an unbiased screen, we developed a cell-based assay that allows simultaneous screening of thousands of shRNAs using FACS and NGS (Fig. 1). THP1 cells were transduced with a heterogeneous mixture of 27,500 barcoded shRNAs included in the DECIPHER™ human module 1 library. Following transduction, shRNA^+^ THP1 cells were infected with a strain of *Mycobacterium bovis* Bacillus Calmette-Guerin (BCG) expressing cyan fluorescent protein (CFP-BCG). Cells permissive for infection, i.e. BCG^high^, cells resistant to infection, i.e. BCG^low^, and uninfected cells were isolated by FACS (Fig. 1A). Cells with low bacterial load included clones where BCG uptake was blocked along with those where BCG had been killed or had defective growth. Re-infection and re-isolation of BCG^low^ cells demonstrated that they were enriched for a mycobacteria-resistant phenotype as evidenced by an increased percentage of BCG^low^ cells with subsequent rounds of selection (Fig. 1B). Since BCG^high^ cells had poor viability when kept in culture for prolonged periods of time, consecutive rounds of selection were not feasible with these cells, and DNA had to be isolated after the first round of selection.

To identify the shRNAs responsible for two phenotypes, integrated shRNA-specific barcodes were amplified by PCR with vector-specific primers and identified using next generation sequencing (NGS) on an Illumina sequencing platform. Uninfected cells were used as control (shRNA^+^/no BCG) to measure the relative level of each shRNA in the transduced cell population without selection. shRNA sequence frequencies in the uninfected controls were compared to that of the BCG^high^ and BCG^low^ cells. Samples yielded 19.2 to 37 × 10^6^ high quality reads with greater than 99% library coverage in uninfected cells and after initial infections, and significant lower coverage (82.42%) after successive rounds of selection (Table 1). The barcode sequence frequency ranges were 6–8, 281 in uninfected control, 1–8, 046 in BCG^high^, 1–107, 809 in BCG^low^ after the second round of selection and 1–268, 377 in BCG^low^ after the third round of selection (Table 1), indicating that selection had occurred in BCG^low^- but not in BCG^high^-cells. Analysis of barcode sequence frequency distributions after log-transformation revealed a normal distribution for uninfected- and BCG^high^-cells and a skewed distribution for BCG^low^ cells, particularly after the third round of selection (Supplementary Fig. S1), providing further evidence for shRNA selection in cells that control BCG infection. Thus, these screening conditions lead to the strong and progressive enrichment of clonal populations of cells carrying shRNAs that induce changes of cell function leading to improved mycobacterial control.

### Oxidoreductases are the most prominent functional group among “hits” in mycobacteria-resistant macrophages

To identify the shRNAs that either blocked uptake or decreased mycobacteria’s adaptability to the intracellular environment, the raw barcode sequences identified in uninfected-, BCG^low^- and BCG^high^- cells were first deconvoluted to obtain the correspondent shRNA sequence lists and their corresponding gene names and annotations. The shRNA sequence frequencies in each sample were then normalized to 10^7^ total reads. “Hits” were defined as shRNA sequences whose frequencies in the experimental sample were increased at least 2-fold over the maximum sequence frequency detected in the uninfected control.

**Figure 2:**
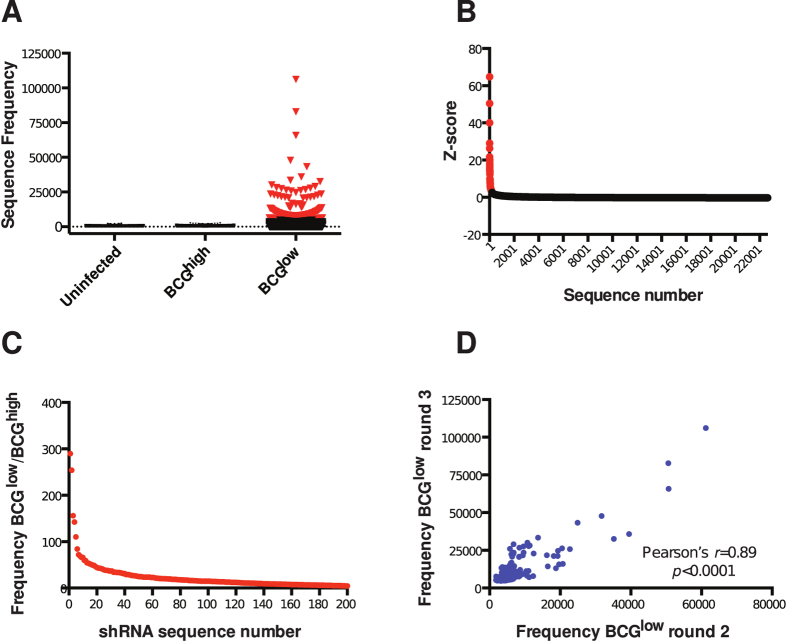
Two hundred and five “hits” identified in cells that resist BCG infection. (**A**) Sequence frequencies in uninfected-, BCG^high^- and BCG^low^- THP1 cells. Each symbol represents one sequence. Red symbols represent 205 sequences that are above the cut off value. (**B**) Distribution of Z-scores of sequences in BCG^low^ cells obtained after the third round of selection. Each symbol represents one sequence. Red symbols represent 290 sequences with Z-scores >2. (**C**) Ratio of frequencies of 205 “hits” between BCG^low^- and BCG^high^ cells. BCG^low^ cells obtained after the third round of selection were used in this calculation. (**D**) Correlation of 205 “hit” frequencies measured in BCG^low^ cells after the second and third rounds of selection.

As expected, none of the sequences in BCG^high^-cells qualified as “hit” due to the lack of shRNA enrichment after a single round of selection. On the other hand, 205 “hits” were identified in BCG^low^ cells, suggesting that the silencing of this discrete set of host genes may favor control of mycobacteria in phagocytes (Fig. 2A, Supplementary Table S1). In BCG^low^ cells, 290 sequences, including all the 205 sequences selected as “hits”, had Z-scores higher than 2 (Fig. 2B). The frequencies of “hits” were increased 3.5- to 290-fold in BCG^low^ cells compared to BCG^high^ cells (Fig. 2C). There was a high degree of correlation between the sequence frequencies detected in BCG^low^ cells after the second and third round of selection, and for most of the 205 “hits” sequence frequency was increased in the last round of selection compared to the previous one (Fig. 2D, Supplementary Table S1).

NGS data obtained with the HiSeq 2500 (Illumina) was highly correlated with data obtained using the Personal Genome Machine (PGM, Ion Torrent™/Life Technologies), which proved to be faster and more cost-effective. Although the total number of sequences detected with the PGM was 60 to 95-fold lower than those obtained with the Illumina platform (Supplementary Table S2), after normalization to total number of reads, the sequence frequencies obtained with the two NGS platforms were comparable and displayed a significant degree of correlation, indicating that the two technologies can be used interchangeably to identify Cellecta barcodes (Supplementary Fig. S2).

Bioinformatics analysis was performed to characterize “hits” identified in BCG^low^ cells. Functional annotation clustering using DAVID bioinformatics database revealed a set of five enriched functional annotation clusters (enrichment scores >2; *p* < 0.01), among which the oxidoreductase cluster had the highest enrichment score with a total of 21 “hits” (Tables 2 and 3). Further analysis of this set revealed that according to the International Union of Biochemistry and Molecular Biology enzyme classification, 18 “hits” in this set are enzymes or components of multi-molecular complexes directly involved in oxidoreduction reactions (class 1, EC 1) and thus participate in the transfer of electrons from a donor to an acceptor. One “hit” (PGC1-α) is a transcriptional co-activator that regulates oxidoreductases. For two “hits” (PIWIL2, SIRT7) no oxidoreductase enzymatic or regulatory activity could be corroborated. Other functional sets and signaling pathways that were either predicted to be involved in mycobacterial uptake or survival (endocytosis, FcγR- mediated phagocytosis, apoptosis, autophagy), or were reported before by other groups (TGF-β signaling pathway) were also found (Supplementary Fig. S3).

**Figure 3:**
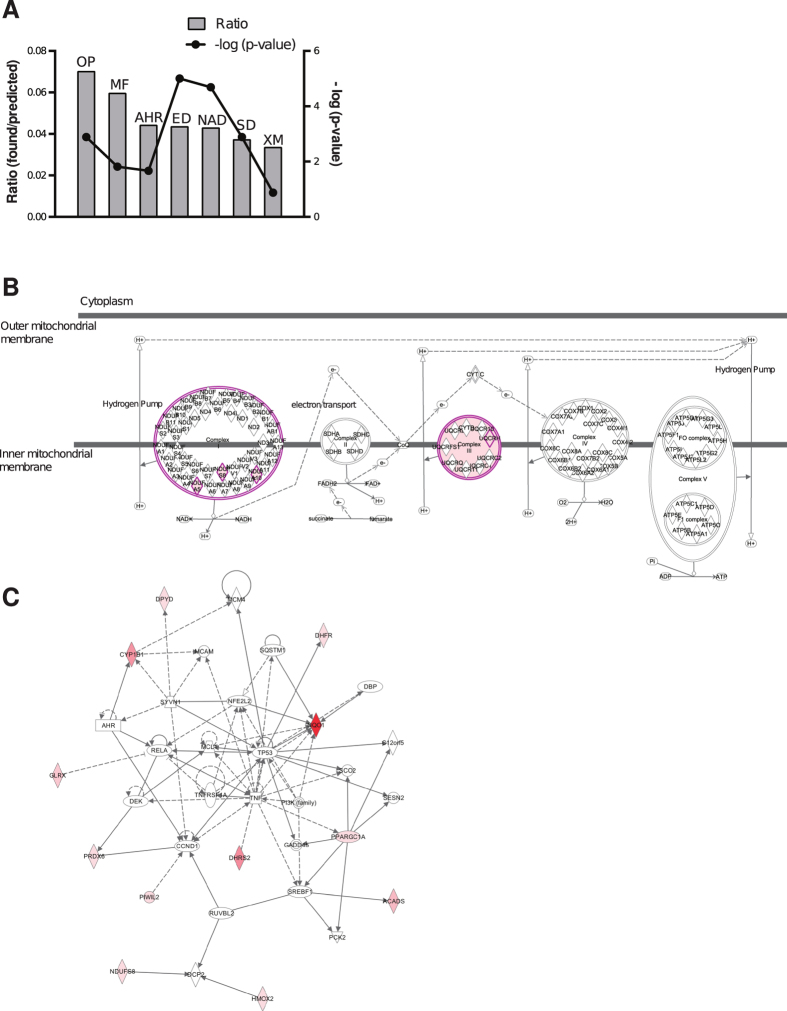
Main signaling pathways and protein networks identified by IPA^®^ within the oxidoreductase functional group enriched in BCG^low^ cells. (**A**) Bar graphs for the highest scoring canonical pathways ranked according to the ratios and statistical significance calculated by Fisher’s exact test right-tailed. The right y-axis displays the -log (p-values) corresponding to the points connected by a thin line. The bars represent the ratios, whose magnitudes are displayed on the left y-axis. Ratios are calculated as follows: number of genes in a given pathway identified as “hits”/total number of genes that make up that pathway in the reference gene set (i.e. human genome). OP = oxidative phosphorylation. MF = Mitochondrial function. AHR = aryl hydrocarbon receptor signaling. ED = ethanol degradation. NAD = noradrenaline and adrenaline degradation. SD = serotonin degradation. XM = xenobiotic metabolism signaling. (**B**) Schematic representation of the oxidative phosphorylation pathway highlighting (purple) the four members identified in the screening with the QIAGEN’s IPA^®^- software (NDUFA5, NDUFA10, NDUFS8, UQCRH). (**C**) The top QIAGEN’s IPA^®^-generated protein network (p < 0.0001) involved in energy production, molecular transport and nucleic acid metabolism includes twelve (red colored symbols) of the twenty-one oxidoreduction “hits” identified in BCG^low^ cells. Direct interactions are depicted with full lines and indirect interactions with dotted lines. The intensity of the red color of the symbols correlates with the level of enrichment of the correspondent shRNA in the screening. White symbols represent molecules that are part of the network but were not identified in the screening.

Host oxidoreductases were not identified in previous siRNA screenings for host factors that regulate intracellular mycobacterial infection. Thus, to start examining the biological relevance of these findings, we further analyzed this set of oxidoreductase “hits” with QIAGEN’s IPA^®^ and identified the following canonical pathways: 1) oxidative phosphorylation (4/104), 2) mitochondrial function (4/165), 3) aryl hydrocarbon receptor signaling (3/135), 4) ethanol degradation II (2/30), 5) noradrenaline and adrenaline degradation (2/32), 6) serotonin degradation (2/52) and 7) xenobiotic metabolism signaling (3/256) (Fig. 3A). Seven of the twenty-one oxidoreductases are mitochondrial enzymes encoded by nuclear genes (ACADS, DMGDH, ETFA, NDUFA5, NDUFA10, NDUFS8 and UQCRH); three of them belonging to complex I (NDUFA5, NDUFA10, NDUFS8), and one to complex III (UQCRH) of the mitochondrial respiratory chain (Fig. 3B). A protein network associated with energy production, molecular transport and nucleic acid metabolism that included 12 of the 21 genes involved in oxidation-reduction identified in our screening was also extracted (p < 0.0001, Fisher’s exact test) (Fig. 3C). These results suggest that mycobacteria exploit host oxidoreductases to regulate energy production, the redox balance and/or detoxification reactions to adapt to the intracellular environment and survive.

### Silencing or chemical inhibition of the top oxidoreductase “hit” NQO1 decreases intracellular mycobacterial survival

**Figure 4:**
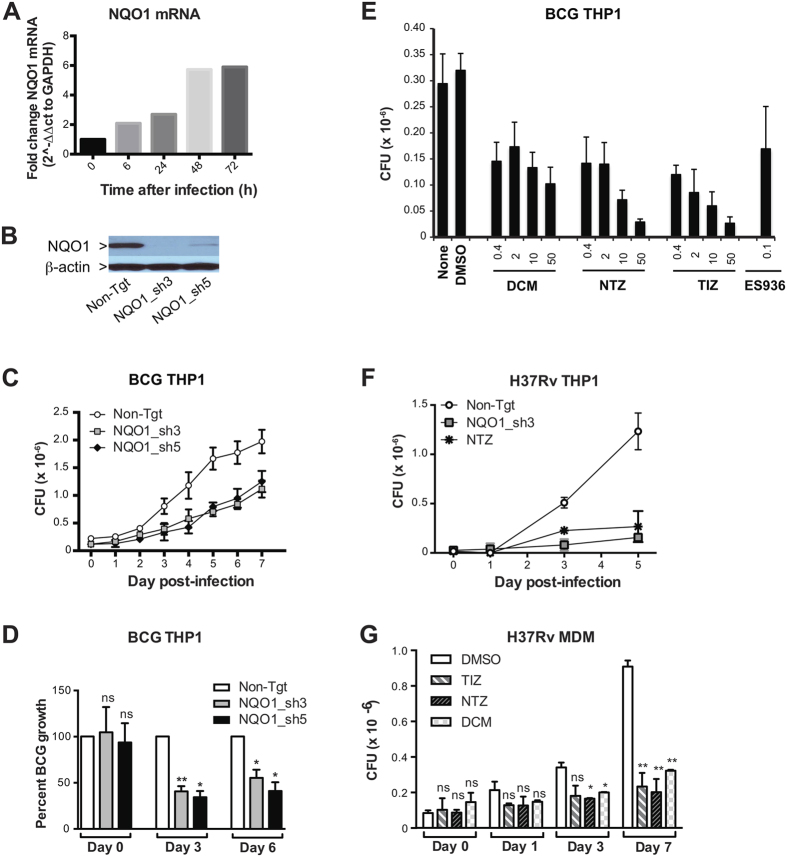
NQQO1 silencing or chemical inhibition decreases intracellular mycobacterial growth. (**A**) NQO1 mRNA was measured in THP1 cells at indicated times after BCG infection by RT-PCR. Shown are relative levels of NQO1 mRNA normalized to GAPDH mRNA of one representative experiment of three. (**B**) NQO1 protein expression was measured in THP1 cells stably expressing non-targeting (Non-Tgt)- or NQO1-shRNAs (NQO1_sh3, NQO1_sh5) by Western blotting. (**C**,**D**) THP1 cells stably expressing non-targeting (Non-Tgt-)- or NQO1-shRNAs (NQO1_sh3, NQO1_sh5) were infected with BCG (MOI 3:1), and CFU were measured in cell lysates at indicated times after infection. Mean CFU ± SD of 6–9 technical replicates/condition from one representative experiment (**C**) and mean percent BCG growth ± SEM of three independent experiments (**D**) are shown. Percent BCG growth = CFU NQO1_sh/CFU Non-tgt ×100. (**E**) THP1 cells were pre-treated with media alone (none), DMSO (0.05%), dicoumarol (DCM), nitazoxanide (NTZ,), its active metabolite tizoxanide (TIZ) or ES936 at indicated concentrations (μM) for 1 h and infected with BCG (MOI 3:1). CFU were determined in cell lysates three days post-infection. Means ± SD of 6–9 technical replicates/condition of one representative experiment of four is shown. (**F**) THP1 cells stably expressing Non-Tgt- or NQO1 (NQO1_sh3)- shRNA or pre-treated with NTZ were infected with H37Rv (MOI 3:1) and CFU measured in cell lysates. (**G**) Primary human monocyte-derived macrophages (MDM) were pre-treated with DMSO, NTZ, TIZ or DCM (10 μM) and infected H37Rv (MOI 3:1) and CFU were measured in cell lysates. Shown are the means ± SD of two independent experiments performed with different donors. Statistical significance was determined by Student’s t test (**D**,**G**) *p < 0.05; **p < 0.01.

To begin to assess the success of the screening, we selected the top “hit” within the oxidoreductase functional cluster, NAD(P)H dehydrogenase, quinone 1 (NQO1), for validation. NQO1 was the most enriched oxidoreductase found in our screening both in terms of absolute frequency and ratio of frequencies between BCG^low^ and BCG^high^ cells (Table 3). NQO1 was also in the top five overall screening “hits” by frequency (Supplementary Table S1). Importantly, NQO1 mRNA expression was induced after mycobacterial infection (Fig. 4A) in a time-dependent manner, suggesting that NQO1 is a target of mycobacterial regulation. NQO1 is a major target of the NF-E2-related factor (Nrf2)/anti-oxidant response element (ARE) signaling pathway, which controls the response to agents that cause oxidative and xenobiotic stress. Nine of 205 “hits” in our screening are part of or regulate the Nrf2/ARE pathway (Table 4). Thus, NQO1 represented an attractive target for validation.

To confirm the role of NQO1 in mycobacterial infection, we used single shRNAs and drug inhibitors to knockdown or functionally inhibit NQO1 in THP1 cells and MDM, and a CFU assay to evaluate intracellular mycobacterial growth. NQO1 knockdown (Fig. 4B) reduced significantly the growth of BCG in THP1 cells (Fig. 4C,D). Dicoumarol and EC936 are well-established NQO1 inhibitors, and nitazoxanide (NTZ) and its metabolite tizoxanide (TIZ) have ben recently described as inhibitors of NQO1^18^. When tested in THP1 cells, all four inhibitiors decreased BCG growth (Fig. 4E). The intracellular growth of the virulent *M. tuberculosis* strain H37Rv was also decreased after NQO1 knockdown and NTZ treatment in THP1 cells (Fig. 4F) and after treatment with dicoumarol, NTZ or TIZ in primary MDM (Fig. 4G). The decrease of intracellular mycobacterial growth observed was not due to decreased bacterial uptake (Supplementary Fig. S4) or drug cytotoxicity, except at the highest concentration of TIZ and NTZ (50 μM) where some cytotoxicity was observed (Supplementary Fig. S5). In agreement with de Carvalho *et al*.^19^ we found that only at high concentrations (≥50 μM) NTZ and TIZ have some inhibitory effect on mycobacterial growth in axenic liquid cultures (MIC of NTZ = 16 μg/ml = 52 μM)^19^ (Supplementary Fig. S6). On the other hand, dicoumarol had no effect on mycobacterial growth in liquid culture at any the concentrations tested (Supplementary Fig. S6). This suggests that the effect of 10 μM NTZ or TIZ and <50 μM dicoumarol on the intracellular mycobacterial growth observed in our cell-based assays, is probably due to an indirect effect on the host cell rather than a direct effect on the bacteria. Altogether this data shows that silencing or chemical inhibition of host NQO1 decreases mycobacterial adaptation to the intracellular environment and suggests that mycobacteria may induce NQO1 to improve their ability to survive and replicate within phagocytes.

### NQO1 inhibition enhances mycobacteria-triggered cell differentiation, NF-κB activation, pro-inflammatory cytokine production and autophagy

NQO1 is a cytosolic two-electron reductase that catalyzes the reduction of quinones and their derivatives to less reactive hydroquinones. These reactions bypass the production of semiquinones, preventing the formation of reactive oxygen species (ROS). In addition, NQO1 can function as direct superoxide scavenger^20^. ROS play a role in *M. tuberculosis* infection via ROS-dependent signaling rather than by direct bacterial killing^21^,^22^. Thus, to begin to define the mechanism by which NOQ1 inhibition leads to decreased mycobacterial growth in macrophages, we first determined if functional inhibition of NQO1 regulates the total levels of ROS in macrophages using the ROS-sensitive dyes DCFH-DA (hydrogen peroxide) and DHE (superoxide) by FACS and fluorescence microscopy. Unexpectedly, we did not find evidence of increased accumulation of ROS in NQO1-knockdown cells compared to non-targeted control after BCG infection or after treatment with the potent ROS inducer pyocyanin (PCN) (Supplementary Fig. S7). Under the microscope, both NQO1-knockdown and control cells treated with PCN exhibited intense cytoplasmic green fluorescence after incorporation of the ROS sensitive dye DCFH-DA (Supplementary Fig. S8). The intense fluorescence exhibited by the PCN-treated cells allowed for a close examination of cell morphology that revealed increased differentiation and spreading, i.e. loss of cell rounding, in NQO1 knockdown cells in response to BCG infection compared to control cells (Supplementary Fig. S8). Also, analysis of cell morphology at higher magnification using brightfield illumination demonstrated a higher frequency of NQO1 knockdown cells with spike-like cellular processes (Supplementary Fig. S8). The percent of non-circular, i.e. fusiform, cells was significantly increased in NQO1 knockdown cells compared to control, as measured by automated fluorescence microscopy (Supplementary Fig. S8).

**Figure 5:**
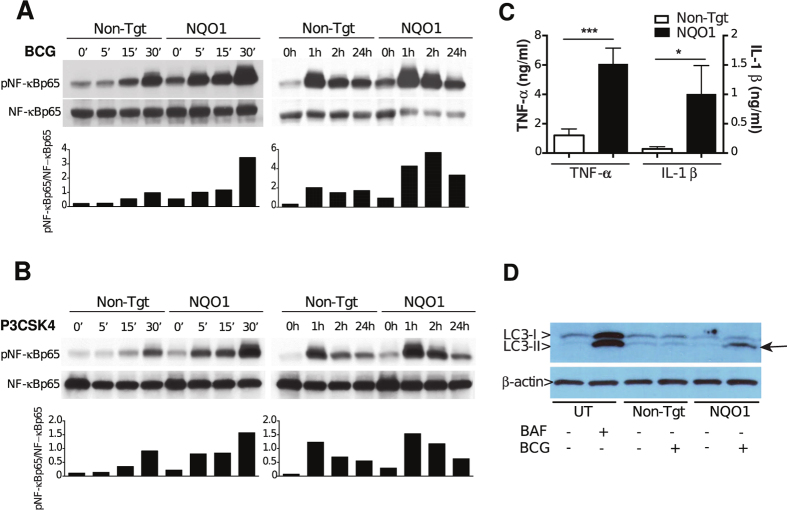
NQO1 deficient cells have increased pro-inflammatory responses to BCG infection. (**A**,**B**) THP1 cells stably expressing non-targeting (Non-Tgt)- or NQO1 (NQO1)- shRNA were infected with BCG (**A**) or stimulated with P3CSK4 (**B**). NF-κBp65 phosphorylation was determined by Western blotting at different time-points post-treatment. The relative optical densities of phospho-NF-κBp65 (p-NF-κBp65) and total NF-κBp65 were measured with ImageJ software. Bar graphs represent the ratios between pNF-κBp65- and NF-κBp65- band optical densities. Representative experiments of a total of three with each stimulus are shown. (**C**) THP1 cells stably expressing non-targeting (Non-Tgt)- or NQO1 (NQO1)- shRNA were infected with BCG and TNF-α and IL1β were measured in 24 h culture supernatants by ELISA. Mean ± SEM of three independent experiments are shown. (**D**) Untransduced THP1 cells were treated with bafilomycin (BAF) and non-targeting (Non-Tgt) or NQO1 (NQO1)- shRNA transduced THP1 cells were infected with BCG and the autophagy marker LC3-II, and LC3-I were detected by Western blotting. One representative experiment of four is shown. Arrow indicates LC3-II accumulation. Statistical significance was determined by Student’s t test (**C**), ***p < 0.001.

Increased cell differentiation in NQO1 knockdown cells was also observed after PMA treatment alone or after BCG infection without PMA pre-treatment, although to a lower extent than with a combination of PMA pre-treatment and BCG infection. Both PMA, through PKC, and mycobacteria, through TLR2, induce NF-κB activation^23^,^24^,^25^, and Nrf2 and NQO1 have been shown to down-regulate NF-κB in different *in vitro* systems^26^,^27^,^28^. We therefore compared NF-κBp65 phosphorylation in NQO1-knockdown and control cells after BCG infection. As shown in Fig. 5, NF-κBp65 phosphorylation was significantly increased as early as 5 minutes after BCG uptake and remained elevated up to 24 h post-infection in NQO1-deficient compared to control cells (Fig. 5A). To test if increased NF-κB activation in NQO1 knockdown cells could be re-capitulated with other known triggers of NF-κB signaling, we used the TLR2 agonist P3CSK4. As seen with BCG, NQO1-knockdown cells have faster and higher NF-κBp65 phosphorylation than control cells in response to TLR2 signaling (Fig. 5B).

In addition to NF-κB activation, the pro-inflammatory response to mycobacteria includes secretion of cytokines such as TNF-α and IL-1β.These cytokines have central roles in mycobacterial infection. In particular TNF-α, directly inhibits intracellular mycobacterial growth^29^. Accordingly, we found significant and pronounced differences in the levels of TNF-α (5-fold) and IL1β (14-fold) secreted by NQO1-knockdown- compared to control cells in response to BCG infection (Fig. 5C).

Since autophagy has emerged as a key anti-mycobacterial host mechanism and NQO1 has been shown to down-regulate autophagy^30^, we tested if the expression of NQO1 in THP1 cells influences autophagy induction by BCG infection. As predicted, increased accumulation of lipidated LC3II, a marker of autophagosomes, was detected in NQO1-knockdown- but not in control cells after BCG infection (Fig. 5D). Altogether, our data indicate that the absence of NQO1 primes cells for accelerated and increased NF-κB activation, readily secretion of TNF-α and IL1β and increased autophagy in response to mycobacterial infection. This suggests that NQO1 functions as a gatekeeper of the NF-κB signaling pathway and cytokine responses and that mycobacteria depend on host NQO1 to down-regulate pro-inflammatory and anti-bacterial functions, resulting in bacterial survival and replication.

### NQO1 inhibition synergizes with antibiotic treatment for intracellular killing of mycobacteria

**Figure 6:**
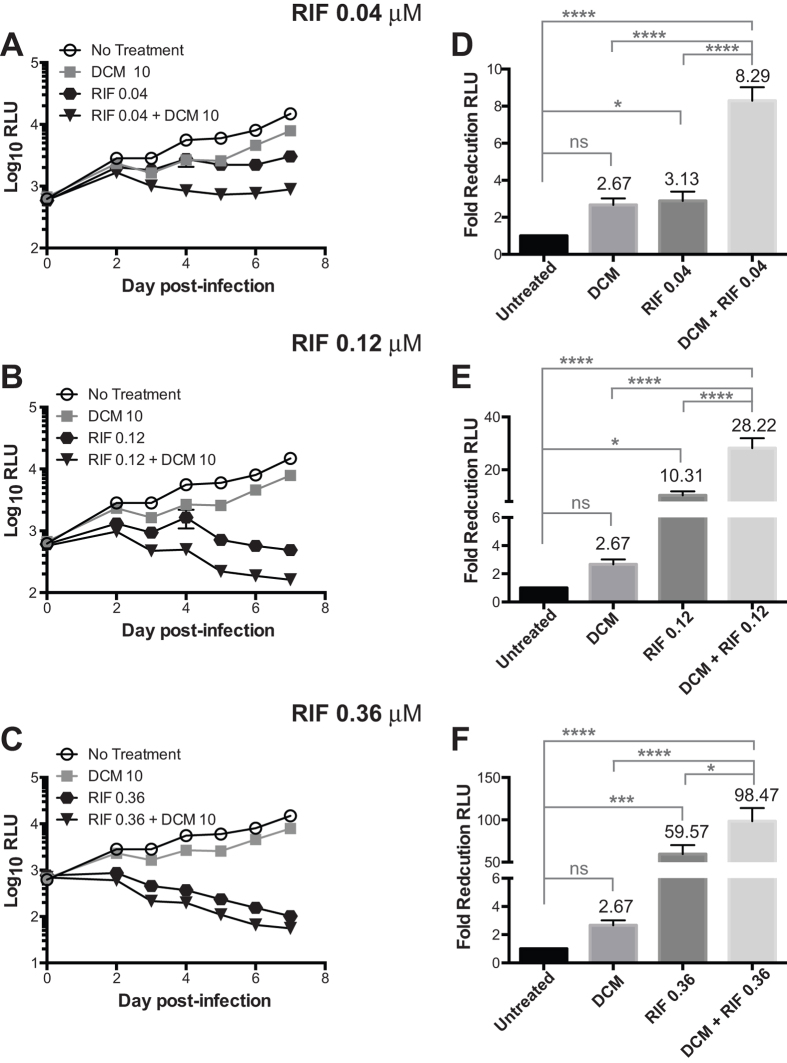
The small molecule NQO1 inhibitor dicoumarol synergizes with rifampin for intracellular mycobacterial killing. (**A**–**C**) THP1 cells were infected with BCG-Lux for 2 h and treated with 10 μM dicoumarol (DCM) alone or in combination with indicated concentrations of rifampin (RIF). Cells treated with media alone served as no treatment control. Intracellular mycobacterial growth was determined by taking daily luminescence readings in a 96-well plate luminometer and expressed as relative luminescence units (RLU). Shown are means ± SEM of 10–12 infections performed in three independent experiments. (**D**–**F**) Fold reduction was determined on day 5 after infection, and represents the number of times the bacterial RLU was decreased in the experimental compared to the untreated control. Fold reduction = 1/(RLU experimental/RLU control). Shown are means ± SEM of 10–12 infections performed in three independent experiments. Two-way ANOVA with the Tukey post-hoc method was carried out to determine significant differences between treatment groups. *p < 0.05, ***p < 0.001, ****p < 0.0001, ns = not significant.

To test the potential of NQO1 as target for HDT, we studied the effects of combining NQO1 inhibition with the first-line anti-TB drug rifampin on the intracellular growth of a luciferase reporter BCG strain^31^. THP1 cells were treated with different concentrations of rifampin (0.04 μM, 0.12 μM and 0.36 μM) and dicoumarol (10 μM) or a combination of both. The rifampin concentrations used in these experiments were determined in advance to be suboptimal for intracellular mycobacterial clearance (IC_50_ = 0.19 μM, Supplementary Fig. S9). Kinetic analysis of intracellular bacterial growth over a period of 7 days indicated that after day 3, cells treated with the drug combination have significantly lower bacterial loads compared to those treated with any of the two drugs alone, especially at rifampin concentrations of or below 0.12 μM (Fig. 6A–C). Furthermore, the fold reduction in RLU with the combined treatment of Rif and dicoumarol exceeded the sum of fold reduction with individual drugs, suggesting a synergistic effect (Fig. 6D–F). Altogether these findings indicate that targeting host NQO1 in combination with antibiotics may produce a synergistic effect that could contribute to faster bacterial clearance in macrophages.

## Discussion

Although many crucial macrophage functions that restrict (e.g. P-L fusion, autophagy, iNOS, ROS, cationic peptides)^13^,^32^,^33^,^34^,^35^ or promote (e.g. lipid body formation)^36^ *M. tuberculosis* growth have been defined, many of the genes that regulate these functions remain to be identified. A broad portfolio of host genes with potential as therapeutic targets is needed for designing HDT and to circumvent the natural attrition of candidates that occurs when compounds are tested *in vitro* and *in vivo*. To this end, we used a large-scale lentiviral-based shRNA screening targeting 5,043 human genes to identify macrophage genes that regulate mycobacterial entry and intracellular survival. While a few RNAi-based large-scale screenings have been reported in mycobacteria-infected macrophages before, ours is the first one using barcoded shRNA-expressing lentiviral vectors in a pooled format. These constructs enabled stable expression of thousands of shRNAs after a single transduction and before bacterial uptake, allowing the study of both early and late infection events. The work presented here identifies NQO1 as a novel host factor required for the optimal survival of mycobacteria in macrophages, sheds preliminary light on the mechanism of this bacterial dependence on NQO1, and establishes that pharmacological inhibition of this novel host target synergizes with a first line antibiotic for intracellular bacterial killing *in vitro*.

Oxidoreductases catalyze the transfer of electrons from reductants to oxidants and participate in many and diverse signaling pathways and cellular functions. Host oxidoreductases as a group have never been linked to the pathogenesis of *M. tuberculosis* infection. Among the cellular functions regulated by the identified set of oxidoreductases are oxidative phosphorylation and mitochondrial function (ETFA, NDUFA5, NDUFA10, NDUFS8, UQCRH)^37^,^38^,^39^, response to oxidative and xenobiotic stresses (AKR1A1, CYP1B1, DHRS2, NQO1, PGC1-α, PRDX6, GLRX)^40^,^41^,^42^,^43^, beta-oxidation of fatty acids (ACADS, ETFA)^44^,^45^, cholesterol- (NSDHL)^46^ and tetrahydrofolate-biosynthesis (DHFR)^47^. In addition, five “hits” in this functional category are involved in the Keap1- Nrf2-ARE stress response pathway including PGC-1α, a transcriptional activator of NRF2^48^,^49^ and two NRF2-target genes (*NQO1*, *AKR1A1*)^50^,^51^,^52^. Due to the diverse nature of the processes they regulate, it is unlikely that all the oxidoreductases identified converge in a single molecular mechanism for regulation of mycobacterial survival. Further work will be required to validate these “hits” and elucidate if host oxidoreductases are hijacked by mycobacteria to decrease uptake or bactericidal functions, or to support metabolic pathways that favor bacterial growth and replication. This may provide not only new insights into the mycobacteria-host interaction but also novel targets for HDT.

NQO1 is a cytosolic flavoenzyme that functions as a two-electron reductase catalyzing the reduction of both xenobiotic and endogenous quinones and their derivatives to less reactive hydroquinones^53^. These reactions bypass the production of semiquinones, preventing the formation of ROS and also contribute to the cycling of endogenous quinones such as ubiquinone and tocopherol quinone^54^. NQO1 can also function as direct superoxide anion radical scavenger^20^,^55^. NQO1 is a major target of the Nrf2/ARE pathway, a master regulator of the anti-oxidant and xenobiotic stress responses^53^. Nrf2/ARE induce detoxifying enzymes and increase cellular energetics and redox potential. Regulation of Nrf2 and NQO1 expression has been reported in *M. tuberculosis* infection *in vivo*^56^. NQO1 has been implicated in the regulation of autophagy, NF-κB, pro-inflammatory cytokines and mitochondrial function^27^,^30^,^57^. NQO1 stabilizes several host proteins including tumor suppressors p53 and p73^58^ and PGC-1α^59^, and is hijacked by HIV-1 to stabilize Tat^60^. Our data shows that NQO1 knockdown increases NF-κB activation, TNF-α and IL-1β secretion and induces autophagy. Increased NF-κB activation in NQO1 deficient cells was observed after BCG infection and following TLR2 stimulation. NF-κB has been shown to promote mycobacterial phagolysosome fusion^61^ and to induce autophagy in a mycobacterial infection model^62^,^63^. TNF-α has a major role in mycobacterial control, both at the cellular level and in the regulation of *in vivo* immune responses^64^,^65^.

We propose a model where NQO1 functions as NF-κB- and TNF-α gatekeeper, and *M. tuberculosis* induces NQO1 expression to inhibit NF-κB, pro-inflammatory cytokines and possibly NF-κB- regulated degradative compartments such as phagolysosomes and autophagosomes. The involvement of other well-known macrophage effector mechanisms including iNOS production^66^,^67^, apoptosis^68^,^69^, vitamin D mediated killing^70^, defensins^71^ and antimicrobial peptides^72^ in regulation of mycobacterial growth by NQO1 is the subject of ongoing studies in our lab.

Other authors have shown that NQO1 loss leads to altered intracellular redox status with an increase in the NADH/NAD^+^ ratio^73^,^74^. Decreased NAD^+^ decreases mitochondrial biogenesis and function via sirtuins (SIRT7 identified in our screening)^75^. Increased NADH may generate reductive stress via two distinct molecular mechanisms^76^ and directly impact mycobacterial metabolism and growth^77^. Nguyen *et al*. have described a novel mycobacterial redox homeostatic system that regulates bacterial NADH levels, i.e. RHOCS, required for *M. tuberculosis* survival in macrophages^77^. Regulation of bacterial RHOCS along with host NQO1 may be a two-pronged strategy for decreasing NADH and reductive stress in mycobacteria. Thus, further work is required to determine if in addition to regulating the NF-κB/pro-inflammatory axis, NQO1 supports *M. tuberculosis’* intracellular survival by regulating NADH/NAD^+^ ratio and/or mitochondrial function.

Due to its high expression in many solid tumors and its role in the reductive activation of anti-cancer agents such as mitomycin C (6-amino-1,1a,2,8,8a,8b-hexahydro-8-(hydroxymethyl)-8a-methoxy-5-methyl-azirino[2′,3′:3,4]pyrrolo[1,2-a]indole-4,7-dione carbamate), E09 (3-hydroxy-5-aziridinyl-1-methyl-2-(1H-indole-4,7-dione)-prop-β-en-α-ol) and β-lapachone (3,4-dihydro-2,2-dimethyl-2H-naphtho(1,2-b)pyran-5,6-dione), NQO1 constitutes an attractive target for selective anti-cancer drug development. In addition, several crystal structures of NQO1 have been solved, including one in complex with dicoumarol, making a structure-based drug discovery approach possible^78^,^79^. NQO1 functions via a “ping-pong” mechanism that relies on sequential two electron transfer from NAD(P)H to FAD to the substrate^80^. Dicoumarol is thought to be the most potent competitive inhibitor of NQO1, vying with NAD(P)H for binding to NQO1 and thus preventing electron transfer to FAD, and has been extensively used to inhibit NQO1 *in vitro* for many decades^81^. Dicoumarol also inhibits vitamin K epoxide reductase (VKOR) preventing recycling of vitamin K and the γ-carboxylation of glutamate residues in clotting factors, which is the basis of its anti-coagulant effect^82^. Interestingly, *M. tuberculosis* expresses a homologue of mammalian VKOR, Rv2968c^83^. However, our data indicates that at concentrations below 50 μM, dicoumarol has no effects on mycobacterial growth in axenic cultures, thus it can be concluded that dicoumarol decreases intracellular mycobacterial survival by acting on the host target. Additionally, the fact that intracellular survival of mycobacteria was decreased with three additional NQO1-targeting drugs, including the mechanism-based inhibitor ES936, suggests that the target of dicoumarol in our assay is probably NQO1 and not VKOR^18^,^84^. Since dicoumarol is unlikely to have value as anti-cancer drug owing to its anticoagulant properties, considerable effort has been made to identify related structures with increased potency of inhibition of NQO1, improved specificity and reduced off-target effects^85^,^86^,^87^. Thus, it will be important to determine the effect of these new NQO1 inhibitors in mycobacterial intracellular survival alone and in combination with first line TB antibiotics.

Here we showed that dicoumarol and rifampin act synergistically to reduce intracellular mycobacterial survival. This suggests that NQO1 inhibition may, in addition to directly induce killing mechanisms in the cell, potentiate the effect of antibiotics by either directing the bacteria to acidic compartments or by altering its metabolic state. A TB therapy based on NQO1 targeting in combination with current first-line antibiotics may contribute to more quickly decreasing the bacterial burden below the spontaneous mutation frequency, thus reducing the probability of developing antibiotic-resistant TB. In addition, NQO1-based combination therapy may be useful for treating antibiotic-resistant strains by making second-line antibiotics more effective.

In summary, we developed a pooled shRNA screening that successfully identified the host oxidoreductase NQO1 as a novel target of *M. tuberculosis* regulation that facilitates intracellular mycobacterial survival. Mycobacteria-dependence on NQO1 is linked to the control of pro-inflammatory and anti-bacterial mechanisms. We also provided proof-of concept demonstration that targeting host NQO1 is not cytotoxic and synergizes with a first-line antibiotic for intracellular mycobacterial killing. These promising results suggest that NQO1 inhibitors, especially compounds with increased specificity and potency, could be developed for adjuvant HDT for TB treatment. Given the limitations of *in vitro* systems to predict *in vivo* drug efficacy, further *in vivo* studies in animal models and humans are warranted to determine if our findings will translate into effective NQO1-based HDT for human TB.

## Materials and Methods

### Ethics statement

Peripheral blood was collected from normal human subjects, in accordance to the protocol approved by Case Western Reserve University’s research ethics institutional review board. Informed written consent was obtained from all participants.

### Bacteria

*Mycobacterium bovis* BCG, Connaught (ATCC 35745, Manassas, VA) was grown in Middlebrook 7H9 medium (BD Diagnostics) supplemented with 10% albumin-dextrose-catalase (ADC) (Difco, Detroit, MI). During mid-log growth, the culture was supplemented with glycerol (6% v/v), divided into aliquots and stored at −80 °C until further use. Bacterial titers were determined by counting colony-forming units (CFU) on 7H10 agar as described^88^. Cyan fluorescent protein-expressing BCG (CFP-BCG) was generated by transformation with plasmid pDH3 (Yeast Resource Center, University of Washington, WA) cloned into pMV262^89^ as described^90^. The auto-luminescent BCG reporter strain expressing the *luxABCDE* operon (BCG-Lux) was generated by transformation with the pMV306hsp+Lux plasmid, a gift from Brian Robertson and Siouxsie Wiles (Addgene #26159), as described^91^. *Mycobacterium tuberculosis -*H37Rv (Colorado State University) was grown on 7H10 agar plates and bacterial colonies were transferred to liquid GAS media and grown to an absorbance of 1.1 (595 nm). Bacterial culture aliquots prepared in media supplemented with 20% glycerol were frozen at −80 °C until use. Stock bacterial counts were determined by the CFU method.

### Antibodies and drug inhibitors

The following Abs were used: mouse anti-NQO1 monoclonal Ab (mAb) (39–3700, Invitrogen), rabbit anti-LC3 polyclonal Ab (pAb, PM036, MBL International Corp.), anti- β-Actin (13E5) rabbit mAb (4970, Cell Signaling), peroxidase-conjugated- goat anti-mouse IgG(H + L) and mouse anti-rabbit IgG(H + L) (115-035-062, 211-035-109; Jackson ImmunoResearch Labs) and alexa fluor-488 goat anti-rabbit IgG (A11008, Invitrogen). Dicoumarol (287897) was from Calbiochem and rapamycin (tlrl-rap) from Invitrogen. ES936 (4022) was purchased from Tocris Bioscience. Nitazoxanide (NTZ, 2-acetyloxy-N-(5-nitro-2-thiazolyl)benzamide) and its active metabolite tizoxanide (TIZ) were kindly provided by Eli Lilly. Dimethyl sulfoxide (DMSO; W387509) and N,N-Dimethylformamide (DMF; 227056), used to dilute small molecule inhibitors and used in cell culture (0.05%) as solvent controls, were purchased from Sigma-Aldrich. All drugs were aliquoted and kept at −80 °C until use. Bafilomycin A1, (B1793) was obtained from Sigma-Aldrich. 3-(4,5-dimethylthiazol-2-yl)-2,5-diphenyltetrazolium bromide (MTT; M-6494) was purchased from Life Technologies, Thermo Fisher Scientific.

### Cell culture

The human monocyte cell line THP1 (ATCC #TIB-202) and primary monocyte-derived macrophages (MDM) were cultured at 37 °C 5% CO_2_ in RPMI 1640 (GIBCO laboratories) supplemented with 10% pooled human serum (SeraCare Life Sciences, Milford, Mass), 20 mM HEPES, 2 mM L-glutamine, 1 mM sodium pyruvate, non-essential amino acids, 100 U/ml penicillin, and 100 μg/ml streptomycin (all from BioWhittaker, Walkersville, MD), unless otherwise indicated.

### Generation of monocyte-derived macrophages (MDM)

Peripheral blood mononuclear cells (PBMC) were isolated from 240cc of blood from six healthy tuberculin skin test negative donors (18–45 year old) recruited among laboratory staff. PBMC were isolated by density gradient centrifugation over sodium diatrizoate/Hypaque (GE HealthCare, Uppsal, Sweden). For monocyte isolation, PBMC were incubated on plastic tissue-culture dishes (Falcon, Becton Dickinson) pre-coated with pooled human serum for 1 h at 37 °C; non- adherent cells were removed, dishes were extensively washed and adherent cells collected by scraping with a plastic policeman. Monocytes were re-suspended in culture media, plated in 96-well plates and differentiated by culturing for additional for 5–7 days before infection.

### shRNA library

The DECIPHER™ shRNA lentiviral human module 1 (HM1) library (Cellecta Inc.) was obtained through the DECIPHER Open Source RNAi Screening Project (http://www.decipherproject.net/) funded by NIH grants CA098374, NCRR RR024095, NHGRI HG003355, NCI CA134062 and NCI CA141848. The DECIPHER™ HM1 library encompasses 27,500 shRNA sequences targeting 5,043 genes (5–6 shRNAs/mRNA), including members of major canonical and non-canonical signaling pathways, 2500 top-ranking genes from the Cancer Genome Atlas and FDA drug targets. Each shRNA was tagged with a unique 18 nucleotide-barcode sequence, which allowed for downstream identification by Illumina NGS. The vector HTS3 cassette (DECIPHER pRSI9-U6-(sh)-HTS3-UbiC-TagRFP-2A-Puro-dW) contains the following elements: (i) U6 RNA polymerase III promoter driving shRNA expression, (ii) 18-nucleotide DNA barcode sequence and (iii) UbiC promoter driving red fluorescence protein (RFP) expression to mark transduced cells, iv) a puromycin selection gene and v) three sets of primers, two for PCR amplification of barcodes and one for DNA sequencing.

### Lentiviral shRNA library packaging

Lentiviral packaging was performed following manufacturer instructions (Cellecta Inc.). The 293T cell line (ATCC™, CRL-11268) was grown in Advanced DMEM (Life Technologies) with 10% FBS and 2 mM GlutaMAX. One day before transfection, 12.5 million cells were plated in a 150-mm tissue-culture dish. On the day of transfection, 60 μg of the plasmid shRNA library was combined with 300 μg of the packaging plasmid mix (Cellecta Inc., psPAX2: pMD2.G) in DMEM without serum or antibiotics in the presence of Plus Reagent™ (1 μl per 1 μg of DNA) and Lipofectamin™ (Life Technologies). Cells were incubated with the DNA/Plus Reagent™/Lipofectamin™ mix for 24 h. Viral supernatant was collected at 48 h and 72 h after transfection, filtered through a 0.45 μm PES filter and concentrated using PEG-it (System Biosciences) per the manufacturer’s protocol. The concentrated lentiviral particles were re-suspended in PBS with 10% FBS and stored at −80 °C.

### Lentivirus transduction

THP1 cells were re-suspended at a density of 2 × 10^5^ cells/ml in DMEM supplemented with 10% FBS and plated in six 15-cm dishes (5 × 10^6^ cells/dish). Each transduction consisted of 1 × 10^8^ cells infected with enough pseudovirus to achieve a 30% efficiency (~3 × 10^7^ infected cells), to ensure that ~90% of the transduced cells were single integrants according to the Poisson distribution. At 24 h post-transduction, media was replaced with fresh DMEM. To generate a purely transduced population, cells were first treated with puromycin (1 μg/ml) for 48 h and then shRNA^+^ RFP^+^ cells were further purified using fluorescence-activated cell sorting (FACS). Cells were then expanded in culture for additional 2–3 days to obtain a starting population (pre-selection) of ≥3 × 10^7^ cells. Before infection with BCG, one cell sample (3 × 10^6^ cells) was harvested and stored as frozen cell pellet to serve as control to measure the relative level of each shRNA in the transduced cell population without selection. The remaining cells were used to perform the screening.

### shRNA screening

The HM1 pooled shRNA lentiviral library was screened in CFP-BCG-infected THP1 cells as summarized in Fig. 1. After viral integration, shRNA^+^ RFP^+^ THP1 cells were infected with CFP-BCG (MOI 3:1) for 24 h, extensively washed and cultured in antibiotic-free media at 37 °C. To maximize the potential for phenotypic effects in the final analysis, we maintained an average representation of >1,000 cells per shRNA construct throughout the screening (i.e. ≥2.7 × 10^7^ cells before each round of BCG infection). In addition, we ensured cells were in log growth at all times during the screening (<70% confluence) to minimize changes in shRNA representation caused by localized restriction of cell growth due to over-confluence^92^. Based on CFP expression detected by flow cytometry, two distinct phenotypes were identified six days after BCG infection in shRNA^+^ cells, BCG^high^ (CFP^high^) cells, representing cells that were permissive for infection, and BCG^low^ (CFP^low^) cells, which were those that were either not infected (defective uptake) or controlled the infection (Fig. 2). BCG^high^ and BCG^low^ cells were then isolated by FACS. Since BCG^high^ cells eventually die in culture due to progressive infection, this phenotype could not be further enriched and cells had to be processed for DNA isolation, PCR and sequencing after the first round of infection. In contrast, BCG^low^ cells were further selected with a second and third rounds of BCG re-infection and re-isolation (Fig. 2). Thus, by repeated challenge and sorting, cells that enable BCG growth were eliminated and the BCG^low^ phenotype enriched. After FACS, cells were collected by centrifugation and the cell pellet was snap-frozen in a dry-ice/ethanol bath. Genomic DNA was purified and the barcodes PCR-amplified and identified by Illumina HT and Ion Torrent^TM^ sequencing.

### Genomic DNA extraction, barcode amplification and next generation sequencing

Genomic DNA was isolated from THP1 cells by using a blood and cell culture DNA mini kit (13323 QIAGEN) as per manufacturer instructions. Pooled barcodes were amplified from 200 μg of genomic DNA by two rounds of PCR using Titanium Taq DNA polymerase mix (Clontech-Takara) and the ABI GeneAmp PCR System 9700 as per the library manufacturer instructions (Cellecta Inc.). The following primers were used for the first round PCR: 5′-TTCTCTGGCAAGCAAAAGACGGCATA-3′ and 5′-TGCCATTTGTCTCGAGGTCGAGAA-3′. For the second round PCR the primers were: 5′-CAAGCAGAAGACGGCATACGAGA-3′ and 5′-AATGATACGGCGACCACCGAGA-3′. PCR products were analyzed by electrophoresis on a 3.5% agarose-1XTAE gel in order to ensure equal yields of amplified barcodes for all samples. Amplified barcodes from 3 × 100 μl second round PCR reactions were combined and DNA was purified using the QIAquick PCR kit (QIAGEN). Products were separated by electrophoresis and DNA isolated from the gel with the QIAquick gel-purification kit (QIAGEN) following the manufacturer’s protocol. DNA was quantified using NanoDrop spectrophotometer (Thermo Fisher Scientific) and concentrations were adjusted to 10 nM. High-throughput sequencing of amplified barcodes was performed at the CWRU Genomics Core on the HiSeq 2500 sequencing platform (Illumina) using the GexSeqN sequencing primer (5′-ACAGTCCGAAACCCCAAACGCACGAA-3′) following the manufacturer’s protocol. Using Illumina Single-Read flow cells, clusters were generated from 20 fmoles (2 μl of 10 nM PCR product) of the gel-purified band from the 2^nd^ round of PCR and the PhiX174 control template. For the HT sequencing step the GexSeq primer was added at a final concentration of 500 nM and 50 cycles of sequencing-by-synthesis (single read) were performed. To verify the sequencing results obtained with the Illumina platform, the same amplicons were used to construct a multiplexed library for shotgun sequencing on the Ion Personal Genome Machine (PGM, Life Technologies). Briefly, barcoded DNA libraries were pooled in equimolar concentrations and templates prepared and enriched for sequencing on the Ion Sphere Particles (ISPs) using the Ion OneTouch 200 Template Kit v2 (Life Technologies) in the Ion OneTouch 2 System (Life Technologies). Templated ISPs were quantified (Qubit 2.0, Life Technologies) and loaded into a Ion 318 Chip (Life Technologies) to be sequenced on the Ion PGM using the Ion PGM Sequencing 200 Kit v2 (Life Technologies). Signal processing and base calling was performed with Torrent Analysis Suite version 3.4.2.

### Sequencing data analysis and “hit” selection

The conversion of raw NGS data into summary files that include annotation for each identified gene and sequence frequencies was done using the barcode deconvoluter software for Mac (Cellecta Inc.). Next, data was processed, edited, normalized, and transformed using Microsoft Excel and GraphPad Prism version 6 for MacOSX (GraphPad Software, San Diego CA). Sequence frequencies in each sample were normalized per 10 million reads. To assess the frequency distribution, read counts per barcode were log- transformed. After normalization, fold enrichment for each sequence in the BCG^low^ sample was calculated over the uninfected control. “Hits” were defined as shRNA sequences whose frequencies in the experimental sample were increased at least 2-fold over the maximum sequence frequency detected in the uninfected control. Then, frequencies of selected sequences were compared between the BCG^low^ and BCG^high^ sample. The standard scores (Z scores) were calculated as follows: z = (x − μ)/σ. “Hits” were clustered in functional categories and signaling pathways using DAVID Bioinformatic Resources 6.7 (NIAID, NIH)^93^,^94^ and QIAGEN’s Ingenuity^®^ Pathway Analysis (IPA^®^, QIAGEN Redwood City, www.qiagen.com/ingenuity).

### NQO1 knockdown with single shRNAs

NQO1 specific (TRCN0000003767, TRCN0000003769, TRCN0000320837)- or control non-targeting -shRNA plasmid DNA vectors (pLKO.1; SHC002, MISSION^®^ shRNAs, Sigma-Aldrich) were packed in self-inactivating viral particles by co-transfection with plasmid pCMV-DR8.91 and envelope vector pMD.G (Addgene) in 293 T cells as per manufacturer instructions. Lentiviral expression constructs were then used to transduce THP1 cells using X-tremeGENE9 DNA transfection reagent (06 365 511 001, Roche) following the manufacturer’s instructions. Off-target effects were tested using three different shRNAs targeting NQO1 and the non-targeting shRNA control constructs in separate transductions. Knockdowns were functionally validated by Western blotting and >70% protein knockdown was considered satisfactory.

### Intracellular bacterial growth assays

THP-1 cells (5 × 10^4^ cells/well) that were either transduced with single NQO1- or non-targeting- shRNA constructs, and THP1 cells or MDM (1 × 10^5^ cells/well) treated with media alone or with NQO1 inhibitors (dicoumarol, NTZ, TIZ, ES936), were infected with BCG, BCG-Lux or H37Rv at MOI of 3:1 in 96-well tissue culture plates. Four hours after infection, cells were extensively washed to eliminate extracellular bacteria and cultured for 0–7 days. Macrophages were observed periodically under the microscope to assess cell viability. The medium was changed on day 4. To determine BCG and H37Rv growth, cells were washed once with phosphate buffered saline (Life Technologies, PBS), lysed in 0.05% SDS and cell lysates plated on 7H10 plates in serial dilutions in 6–9 replicates/dilution. Bacterial colonies were counted after incubation at 37 °C for 2 weeks. Values were expressed as CFU/well. CFU determined immediately after the infection (day 0) served as a measure of bacterial uptake. For testing the effects of combined dicoumarol and rifampin against bacteria residing inside macrophages, we used a 96-well luciferase assay developed by Andreu *et al*.^31^. The assay has been validated and compared to CFU for testing effects of antibiotics against intracellular bacteria. Comparisons of log EC_90_ for several drugs against intracellular bacteria show no significant differences between the results obtained by CFU counts and by bioluminescence readings^31^. However, the bioluminescence assay is considerably simpler and with much shorter turnaround than CFU, allowing the simultaneous study of numerous variables, including drug dosages and culture time-points in significantly less time. Briefly, cells were cultured in 96-well tissue culture white plates with clear bottoms (Corning^®^), infected as above and bioluminescence was measured for 10 s using the GloMax^®^ microplate luminometer (Promega) daily after infection. Bioluminescence was expressed as relative light units (RLU). Wells with uninfected cells served as negative controls.

### Determination of drug direct effects on mycobacterial growth in axenic cultures

BCG were cultured in Middlebrook 7H9 media (Difco) supplemented with 10% BBL Middlebrook OADC enrichment, 0.2% glycerol and 0.05% Tween 80 to mid-log phase (OD_600_ = 0.6–0.7), then centrifuged and diluted to OD_600_ 0.1 in fresh 7H9 Middlebrook media. Bacterial cultures were then treated with or without dicoumarol, NTZ or TIZ (0.4–50 μM) and their absorbance (OD_600_) determined daily for 7 days.

### Cell toxicity assay

Cell viability was determined with the 3-[4,5-dimethylthiazol-2-yl]-2,5 diphenyl tetrazolium bromide (MTT; M2128 Sigma-Aldrich) as described^95^. Alternatively, cell viability after drug treatments was evaluated by cell labeling with Hoescht 33342 fluorescent stain (Thermo Fisher Scientific, 62249) and enumeration of intact nuclei by automated fluorescence microscopy.

### qRT-PCR

Total RNA was extracted from THP1 cells with RNeasy kit (QIAGEN, 74104). Quantification of NQO1 mRNA was performed as described^96^. NQO1-specific primers (PPHO1546C) were from QIAGEN. NQO1 mRNA levels were normalized to the β-actin level using the equation 2^−ΔΔCt^ as described by Applied Biosystems. The level of NQO1 mRNA in uninfected cells was used as reference sample.

### Detection of intracellular ROS

Intracellular ROS levels were measured using the ROS-sensitive dyes DCFH-DA and DHE as described^97^. THP1 cells were differentiated with PMA (20 nM) for 48 h, rested for 24 h and treated with media alone or drugs (1 h) and/or infected with BCG (MOI 3) in 24-well plates or 8-well chamber μ-slides (80826; Ibidi). Cells were then incubated with 2.5 μM CM-H2DCFDA (flow cytometry and microscopy) or with 10 μM DHE (flow cytometry) in HBSS or Krebs-Hepes buffer respectively for 30 min at 37 °C in 5% CO_2_. PCN (10009594; Cayman Chemical Company) was used as a positive control for the induction of ROS. The oxidative conversion of cell-permeable DCFH-DA and DHE to their fluorescent derivatives, indicators of hydrogen peroxide and superoxide production respectively, was determined by flow cytometry using a BD LSR Fortessa. DCFH-DA conversion was also determined by fluorescence microscopy in cells counterstained with Hoescht 33342 fluorescent nuclear stain. Samples were analyzed by fluorescence microscopy in a Cytation 3 automated digital microscope equipped with DAPI and GFP imaging cubes, a 16-bit gray scale, Sony CCD, 1.2 megapixel camera (Biotek, Winooski, VT) and a 10x objective. Nine fields per well were photographed every 15 minutes and images analyzed with the Gen 5 2.06 software (Biotek). The percentage of ROS positive cells were calculated as follows: number of green fluorescent cells/number of blue fluorescent cells ×100. The mean GFP fluorescence was automatically calculated after background subtraction.

### Analysis of cell morphology in cells treated with PCN and DCFH-DA

Using DAPI and GFP imaging cubes and a 10x objective, a total of 9 images/well were captured and analyzed with the Gen5 ver 2.06 software. A mask was created in the blue channel (DAPI) to calculate the total number of cells. In the green channel, a mask was set to allow the enumeration of cells based on GFP (cytosolic) labeling. Within the GFP mask, the number of non-circular cells i.e. with circularity index <0.3 was calculated. Percent of fusiform cells was calculated as: number of non-circular cells/total number of cells ×100.

### Cytokine quantification

Cytokines were measured with the TNF-α Quantikine ELISA (DTA00C) and the human IL-1β Quantikine ELISA (DLB50) kits (R&D Systems, Inc.) following manufacturer’s instructions.

### Assessment of NF-κBp65 phosphorylation

For analysis of NF-κBp65 phosphorylation, cell lysates obtained from uninfected- or BCG-infected NQO1 knockdown or control cells were re-suspended in 2× sample buffer and heated to 95 °C. Proteins (20 μg/well) were separated by SDS-PAGE on 10% Tris-Glycine gels (Novex, EC6078BOX; Life Technologies) under reducing conditions and electro-transferred to 0.2 μm nitrocellulose membranes (162-0112; Bio-Rad, Hercules, CA, USA). After transfer, membranes were incubated at room temperature for 1 h in blocking buffer (1× PBS, 5% BSA and 0.1% Tween-20). Blots were incubated with primary rabbit anti-phospho-NF-κBp65 (Ser536) (3033; Cell Signaling) or mouse anti- NF-κBp65 (6956; Cell Signaling) mAbs followed by peroxidase-conjugated mouse anti-rabbit or goat anti-mouse secondary antibodies (Jackson Immuno Research) for 16 h and 1 h, respectively. Western blots were analyzed with ImageJ software (NIH).

### Autophagy detection

For monitoring LC3 conversion by Western blotting, 2 × 10^5^ THP1 cells/well were plated in 12-well dishes and were infected with BCG at an MOI 3. After 4 h, the infected monolayer was washed extensively with PBS and cells cultured for additional 24 h at 37 °C. As positive control of LC3II accumulation, cells were treated with Bafilomycin A. Cell lysates were prepared and protein samples were resolved in 4–20% Tris-Glycine gels (EC6028, Invitrogen) and electroblotted onto polyvinyl fluoride membrane (Millipore IPVH20200) with 0.2 μm pores, which were then fixed with 0.2% glutaraldehyde in PBS with 0.02% Tween-20 for 20 min at room temperature, prior to blocking with 5% non-fat milk. Blots were incubated with primary antibodies (anti-LC3, anti-β actin) in 5% non-fat dry milk in TBS plus 0.1% Tween20 (Sigma-Aldrich, P1379) overnight at 4 °C. Detection was done with horseradish peroxidase-conjugated anti-rabbit (715-035-150) and anti-goat (705-035-147) secondary antibodies (Jackson Immunoresearch), and visualized with SuperSignal™ West Pico (Thermo Scientific, 34078).

### Statistical analyses

GraphPad Prism version 6 for MacOSX (GraphPad Software, San Diego CA) was used to test for differences in means between specific treatment groups. The null hypothesis of no difference in means between specific treatment groups was tested using a two-tailed Student’s *t-*test. Two-way ANOVA with post-hoc Tukey for multiple comparisons was used for pairwise comparison of multiple treatments. A p-value < 0.05 taken as evidence of a significant difference between groups (*p < 0.05; **p < 0.01; ***p < 0.001; ****p < 0.0001).

## Additional Information

**How to cite this article**: Li, Q. *et al*. Novel high throughput pooled shRNA screening identifies NQO1 as a potential drug target for host directed therapy for tuberculosis. *Sci. Rep.* **6**, 27566; doi: 10.1038/srep27566 (2016).

## Supplementary Material

Supplementary Information

Supplementary Information

## Figures and Tables

**Table 1:** Number of barcodes and range of sequence frequencies identified in uninfected-, BCG^high^ and BCG^low^ THP1 cells.

Condition	Number of reads*	Percent of sequences Q score >30	Number of barcodes identified*	Number of single shRNAs	Range of sequence frequencies
Uninfected	53.9	68.1	35.02	27,495	6–8, 281
Sort 1_BCG^high^	36	86.4	29.9	27,483	1–8, 046
Sort 2_BCG^low^	31	61.9	17.61	24,502	1–107, 809
Sort 3_BCG^low^	33.6	84.7	25.3	22,666	1–268, 377

^*^(×10^−6^).

**Table 2:** Functional annotation clusters identified by DAVID bioinformatics resource among “hits” enriched in cells that resist BCG infection.

Category	Term	Count	*P*-value
**Annotation Cluster 1**	**Enrichment Score: 3**.**33**		
SP_PIR_KEYWORDS	Oxidoreductase	18	7.0 × 10^−5^
GOTERM_BP_FAT	Oxidation reduction	21	4.7 × 10^−4^
GOTERM_MF_FAT	Electron carrier activity	10	3.2 × 10^−3^
**Annotation Cluster 2**	**Enrichment Score: 2**.**92**		
GOTERM_MF_FAT	Protein dimerization activity	20	1.4 × 10^−4^
GOTERM_MF_FAT	Protein homodimerization activity	13	2.0 × 10^−3^
GOTERM_MF_FAT	Identical protein binding	18	6.2 × 10^−3^
**Annotation Cluster 3**	**Enrichment Score: 2**.**67**		
GOTERM_CC_FAT	Membrane-bounded vesicle	17	1.1 × 10^−3^
GOTERM_CC_FAT	Cytoplasmic membrane-bounded vesicle	16	2.2 × 10^−3^
GOTERM_CC_FAT	Vesicle	18	2.4 × 10^−3^
GOTERM_CC_FAT	Cytoplasmic vesicle	17	3.8 × 10^−3^
**Annotation Cluster 4**	**Enrichment Score: 2**.**61**		
GOTERM_BP_FAT	Inflammatory response	14	5.3 × 10^−4^
GOTERM_BP_FAT	Defense response	18	4.6 × 10^−3^
GOTERM_BP_FAT	Response to wounding	16	6.2 × 10^−3^
**Annotation Cluster 5**	**Enrichment Score: 2**.**11**		
GOTERM_BP_FAT	RNA biosynthetic process	13	7.8 × 10^−4^
GOTERM_BP_FAT	Transcription, DNA-dependent	12	2.3 × 10^−3^
GOTERM_BP_FAT	Transcription from RNA polymerase II promoter	10	4.9 × 10^−3^

**Table 3:** “Hits” belonging to the oxidation-reduction functional cluster identified with DAVID bioinformatics resource.

Gene Symbol	Definition	Frequency BCG^low^	Frequency BCG^high^	BCG^low/^BCG^high^
NQO1	NAD(P)H dehydrogenase, quinone 1	43266	602	71.9
DHRS2	Dehydrogenase/reductase (SDR family) member 2	22344	324	68.9
CYP1B1	Cytochrome P450, family 1, subfamily B, polypeptide 1	21146	456	46.4
ACADS	Acyl-Coenzyme A dehydrogenase, C-2 to C-3 short chain	15638	370	42.2
GLRX	Glutaredoxin (thioltransferase) GRX1	13630	417	32.7
DMGDH	Dimethylglycine dehydrogenase	11657	373	31.3
AKR1A1	Aldo-keto reductase family 1, member A1 (aldehyde reductase)	11551	674	17.1
IFI30	Interferon, gamma-inducible protein 30	11146	557	20.0
NSDHL	NAD(P) dependent steroid dehydrogenase-like (NSDHL)	9820	626	15.7
PIWIL2	Piwi-like 2 (Drosophila) (PIWIL2)	9787	757	12.9
NDUFS8	NADH dehydrogenase (ubiquinone) Fe-S protein 8, 23 kDa	8713	167	52.3
DHFR	Dihydrofolate reductase	8530	1491	5.7
DPYD	Dihydropyrimidine dehydrogenase	8387	528	15.9
NDUFA5	NADH dehydrogenase (ubiquinone) 1 alpha subcomplex, 5, 13 kDa	8011	931	8.6
HMOX2	Heme oxygenase (decycling) 2 (HMOX2)	6166	537	11.5
PPARGC1A	Peroxisome proliferator-activated receptor gamma, coactivator 1α	5944	642	9.3
NDUFA10	NADH dehydrogenase (ubiquinone) 1 alpha subcomplex, 10, 42 kDa	5442	791	6.9
ETFA	Electron-transfer-flavoprotein, alpha polypeptide	5362	1013	5.3
PRDX6	Peroxiredoxin 6	5160	822	6.3
SIRT7	Sirtuin (silent mating type information regulation 2 homolog) 7 (S. cerevisiae)	5058	979	5.2
UQCRH	Ubiquinol-cytochrome c reductase hinge protein	4930	1172	4.2

**Table 4:** Hits associated with the NRF2/ARE-mediated oxidative stress response.

Symbol	Gene Name	Frequency BCG^low^	Frequency BCG^high^
NQO1	NAD(P)H dehydrogenase, quinone 1	43266	602
CDC34	Cell division cycle 34	21676	196
AKR1A1	Aldo-keto reductase family 1, member A1	11551	674
CREBBP	CREB binding protein	11043	300
PRKD3	Protein kinase D3	8447	1166
HRAS	Harvey sarcoma viral oncogene homolog	6870	859
MAP2K5	Mitogen-activated protein kinase 5	6395	433
PGC1α	Peroxisome proliferator-activated receptor gamma, coactivator 1 alpha	5944	642
MAP2K6	Mitogen-activated protein kinase 6	4851	1365
