# Supplementary material for: Novel high throughput pooled shRNA screening identifies NQO1 as a potential drug target for host directed therapy for tuberculosis
Source: Sci Rep. 2016 Jun 14;6:27566. doi: 10.1038/srep27566 (PMC4906352; doi:10.1038/srep27566)
Supplement: Supplementary Information [file srep27566-s1.pdf]

Supplementary information

Novel high throughput pooled shRNA screening identifies NQO1 as a potential drug target for host directed therapy for tuberculosis

Qing Li[1], Ahmad F. Karim[2], Xuedong Ding[1], Biswajit Das[2], Curtis Dobrowolski[2], Richard M. Gibson[3], Miguel E. Quiñones-Mateu[3,4,5], Jonathan Karn[2,4] and Roxana E. Rojas[2,4*]

[1] Department of Medicine, Case Western Reserve University & University Hospitals, Cleveland, Ohio, United States of America

[2] Department of Molecular Biology and Microbiology, Case Western Reserve University & University Hospitals, Cleveland, Ohio, United States of America

[3] University Hospitals Translational Laboratory, University Hospitals Case Medical Center, Cleveland, Ohio, United States of America

[4] Center for AIDS Research (CFAR), Case Western Reserve University & University Hospitals, Cleveland, Ohio, United States of America

[5] Department of Pathology, Case Western Reserve University & University Hospitals, Cleveland, Ohio, United States of America

*Corresponding author

E-mail: rxr38@case.edu (RER)

Additional discussion points about the pooled lentiviral shRNA screening

Systematic genetic or chemical approaches for identifying host factors required for bacterial survival and replication have been limited[1-5]. Large-scale RNA interference (RNAi) screens to study bacterial infection were first performed in *D. melanogaster* cells infected with *L. monocytogenes*, *Chlamydia spp*. and *Mycobacterium fortuitum*[1,6,7]. A human kinase silencing RNA (siRNA) sub-library was screened in a human cell line to identify kinases required for intracellular growth of *S. typhimurium*[8]. A genome-wide RNAi screen in human cells have identified intracellular networks regulating *M. tuberculosis* survival[3]. Thus, high-throughput RNAi has emerged as a powerful molecular screening tool for studies of the host-pathogen interactions in different infection models[9]. These previous large-scale screens used siRNA libraries constructed with chemically synthesized oligonucleotides and required massive parallel screening of thousands of individual siRNAs. This approach is expensive and time-consuming, and results are difficult to reproduce. Lentiviral-based libraries containing heterogeneous mixtures of short hairpin RNAs (shRNA) synthesized enzymatically from cDNAs do not require access to expensive HT equipment and can be performed in a single experiment. Pooled shRNA library screens offer several advantages over siRNA: they are fast, efficient and inexpensive, result in deeper coverage and require minimal manipulation of cell cultures, resulting in more precise identification of targets; since shRNAs are transfected into cells via viral vectors (lentiviruses), they integrate into the cellular DNA resulting in stable knockdown of mRNAs, allowing for enrichment of the specific phenotype through multiple rounds of selection.

With one exception[1], in all the RNAi screens reported before, host gene silencing was performed after mycobacterial uptake[2,3,8]. A major advantage of lentiviral-based shRNA targeting is the long-term silencing of host genes that allow for bacterial infection

of cells in which gene expression down-regulation has already occurred. Thus, lentiviral-based shRNA allows for identification of host factors that play a role in the very early events leading to mycobacterial adaptation to the intracellular environment.

Since each RNAi screening is unique in its design, we reasoned different molecules could be identified using different approaches and this could widen the range of host targets for HDT. Although the lack of reproducibility of results of RNAi screens has been criticized, this may not be necessarily a weakness, since each screen may contribute new sets of validated targets to the pool of human molecules known to play a role in the mycobacteria-host interaction. Variability in results among different screens may depend on differences in both experimental set up (bacterial strain, infection protocol, transduction versus transfection, cell viability, etc.) and data analysis, and does not necessarily call into question the validity of the results but reveals the complexity of factors that play a role in the final outcome of mycobacterial infection. Careful validation of screening “hits” with different cell types, bacterial strains and different functional inhibition approaches (e.g. genetic vs. chemical) increases the reliability of screens.

The shRNA screening strategy used in this study was designed to identify host gene products that support mycobacterial uptake, replication and/or survival whose silencing or inhibition contributes to mycobacterial clearance in macrophages. However, another important application for this shRNA screening could be the identification of new host targets whose silencing or inhibition increase intracellular mycobacterial replication or survival. These host molecules are predicted to be involved in innate killing mechanisms or to negatively regulate other host factors required for mycobacterial survival. Drug agonists of these targets may improve mycobacterial control by macrophages. Since several rounds of selection are not feasible in heavily infected cells due to viability constrains, identification of shRNAs that increase mycobacterial growth in macrophages will require modifications of the current screening strategy. The use of

lower MOIs, a first-step cell sorting immediately after infection, shorter culture times before selection and the use of more selective cell sorting gates (very high-, high- and low-CFP) may allow the isolation of cell clones carrying these type of shRNAs and their correspondent host genes.

Literature cited in Supplementary Information

1 Philips, J. A., Rubin, E. J. & Perrimon, N. Drosophila RNAi screen reveals CD36 family member required for mycobacterial infection. *Science* **309**, 1251-1253, doi:10.1126/science.1116006 (2005).

2 Jayaswal, S. *et al.* Identification of host-dependent survival factors for intracellular Mycobacterium tuberculosis through an siRNA screen. *PLoS Pathog* **6**, e1000839, doi:10.1371/journal.ppat.1000839 (2010).

3 Kumar, D. *et al.* Genome-wide analysis of the host intracellular network that regulates survival of Mycobacterium tuberculosis. *Cell* **140**, 731-743, doi:10.1016/j.cell.2010.02.012 (2010).

4 Stanley, S. A. *et al.* Identification of host-targeted small molecules that restrict intracellular Mycobacterium tuberculosis growth. *PLoS Pathog* **10**, e1003946, doi:10.1371/journal.ppat.1003946 (2014).

5 Sundaramurthy, V. *et al.* Integration of chemical and RNAi multiparametric profiles identifies triggers of intracellular mycobacterial killing. *Cell Host Microbe* **13**, 129-142, doi:10.1016/j.chom.2013.01.008 (2013).

6 Agaisse, H. *et al.* Genome-wide RNAi screen for host factors required for intracellular bacterial infection. *Science* **309**, 1248-1251, doi:10.1126/science.1116008 (2005).

7 Prudencio, M. & Lehmann, M. J. Illuminating the host - how RNAi screens shed light on host-pathogen interactions. *Biotechnol J* **4**, 826-837, doi:10.1002/biot.200900071 (2009).

8 Kuijl, C. *et al.* Intracellular bacterial growth is controlled by a kinase network around PKB/AKT1. *Nature* **450**, 725-730, doi:10.1038/nature06345 (2007).

9 Hong-Geller, E. & Micheva-Viteva, S. N. Functional gene discovery using RNA interference-based genomic screens to combat pathogen infection. *Curr Drug Discov Technol* **7**, 86-94 (2010).

Supplementary figure legends

Supplementary Fig. S1. Frequency distribution of barcode (shRNA) sequences identified by Illumina sequencing in uninfected- and BCG-infected THP1 cells. Raw HT sequencing data from uninfected- **(A),** BCG^{high}-**(B)**, BCG^{low} after second selection-**(C)** and BCG^{low} after third selection-**(D)** samples were converted into summary files of annotated sequences. Sequence frequencies were normalized to 10^7 reads and log-transformed before graphing.

Supplementary Fig. S2. **Frequencies for the top 205 sequences identified in BCG^{low} cells: comparison between Illumina and Ion Torrent^{TM} sequencing.** DNA purified from BCG^{low} cells after the third round of selection was amplified with barcode-specific primers and sequenced on the Illumina HiSeq 2500 sequencer and the Ion Torrent^{TM} PGM. The frequencies of the 205 "hits" obtained with each sequencing platform were plotted against each other and the correlation coefficient calculated. Each symbol represents one sequence.

Supplementary Fig. S3. **TGF-β signaling pathway is enriched among "hits" identified in BCG^{low} cells**. Ingenuity pathway analysis (IPA®) identified the TGF-β signaling pathway as significantly enriched in BCG^{low} cells ($p < 0.001$). TGF-β signaling pathway molecules present in the dataset of 205 "hits" are highlighted in pink. Double-bordered molecules correspond to groups or complexes.

Supplementary Fig. S4. **BCG and H37Rv uptake in THP1 cells and MDM after NQO1 knockdown or treatment with NQO1 inhibitors. A.** THP1 cells stably

expressing non-targeting (Non-Tgt)- or NQO1 (NQO1_sh3, NQO1_sh5) -shRNAs were infected with BCG (MOI 3:1) for 4 h and extensively washed to remove extracellular bacteria. CFU were measured in cell lysates immediately after infection. Mean CFU ± SEM of three independent experiments are shown. **B.** Primary MDM were pre-treated with DMSO, NTZ, TIZ or DCM (10 μM) and infected H37Rv (MOI 3:1) for 4 h and extensively washed to remove extracellular bacteria. CFU were measured in cell lysates immediately after infection.

Supplementary Fig. S5. Cell viability after treatment with NQO1 inhibitors. A-B. THP1 cells were treated with indicated concentrations of dicoumarol, NTZ, TIZ or ES936 and cell viability was assessed with the MTT assay on days 1 and 5 after beginning of treatment. Percent cell viability was calculated as follows: OD_{570nm} cells treated with drug/ OD_{570nm} cells treated with DMSO (0.05%) x 100. Shown are means ± SEM of three independent experiments. **C-D**.THP1 cells were pre-treated with medium alone (None), DMSO (0.05%) or different concentrations of NTZ, and then either left uninfected or infected with BCG at MOI 3:1 or 10:1. Cell viability was determined with the MTT assay on days 1 and 5 post-infection. Means ± SD of triplicate samples of one representative experiment of two are shown.

Supplementary Fig. S6. Effect of NQO1 chemical inhibitors on BCG growth in axenic liquid cultures. BCG in 7H9 medium was exposed to different concentrations of dicoumarol (DCM), NTZ or TIZ. The absorbance (OD_{600}) of the bacterial culture was determined at different intervals after initiation of the culture.

Supplementary Fig. S7. **Reactive oxygen species (ROS) levels in NQO1-deficient- and control cells. A-D.** THP1 cells stably expressing non-targeting (Non-Tgt)- or NQO1

(NQO1) -shRNAs were infected with BCG for indicated periods of time (A-B) or treated with pyocyanin (PCN) for 30 min (C-D). Cells were then incubated with 2.5 μM DCFH-DA (B, D) or with 10 μM DHE (A, C) for 30 min at 37°C in 5% CO_2. The oxidative conversion of cell-permeable DCFH-DA and DHE to their fluorescent derivatives, indicators of hydrogen peroxide and superoxide production respectively, was determined by flow cytometry and expressed as mean fluorescence values (MFI)**.** Shown are means ± SEM of three independent experiments. **E-F.** NQO1 knockdown (NQO1) or control (Non-Tgt) THP1 cells were treated with PCN for 30 min in 8-well chamber μ-slides. DCFH-DA conversion was determined by fluorescence microscopy in cells counterstained with Hoescht fluorescent nuclear stain. Samples were analyzed in a Cytation 3 automated digital fluorescence microscope equipped with DAPI and GFP imaging cubes, a 16-bit gray scale 1.2 megapixel camera and a 10x objective. Nine fields per well were photographed every 15 minutes after DCFH-DA treatment and images analyzed with the Gen 5 2.06 software (Biotek). The percentages of ROS positive cells were calculated as follows: number of green fluorescent cells/number of blue fluorescent cells x 100 (E). After background subtraction, the mean GFP fluorescence was automatically calculated by the software (F). Shown are means ± SEM of three independent experiments.

Supplementary Fig. S8. NQO1 deficient cells exhibit increased cell spreading and differentiation. A-B. THP1 cells stably expressing non-targeting (Non-Tgt)- or NQO1 (NQO1)-shRNA were stimulated with pyocyanin (PCN), labeled with DCFH-DA and counterstained with Hoescht 33342 fluorescent nuclear stain. Cells were analyzed by fluorescence microscopy using a 10x objective (A) or under brighfield illumination using a 40x objective (B) in a Cytation 3 automated digital microscope. Images were acquired

with a built in 16-bit gray scale Sony CCD camera. **C**. Using DAPI and GFP imaging cubes and a 10x objective, a total of 9 images/well were captured and analyzed with the Gen5 ver 2.06 software. A mask was created in the blue channel (DAPI) to calculate the total number of nuclei, i.e. cells. In the green channel another mask was set to allow the enumeration of cells based on GFP (cytosolic) labeling. Within the GFP mask, the number of non-circular cells i.e. with circularity index < 0.3, was calculated. Percent of fusiform cells was calculated as: number of non-circular cells/ total number of cells x 100. Shown are means ± SEM of three independent experiments.

Supplementary Fig. S9. Assessment of rifampin intracellular anti-mycobacterial activity by automated hi-res fluorescence microscopy. THP1 cells (35,000/well) were infected with GFP-BCG (MOI 3:1) and left untreated or were treated with different concentrations of rifampin (0.05 -30.4 μM) and cultured for 3 or 7 days. Cells were labeled with CellTrackerRed™ before imaging. Images were acquired on a Leica 6000 B automated inverted fluorescence microscope with a 20x objective connected to a Retiga EXI camera (Q-imaging Vancouver British Columbia). A full-well scanning protocol controlled by Metamorph imaging software was used. Acquired images were analyzed with customized software (ImageIQ, Inc.). **A-B**. Raw images were background corrected and each fluorescence channel enhanced independently A cell mask was then created using the cell tracker channel and then only segmented bacteria within the cell mask were counted. **C-F.** Dose-response curves of log transformed rifampin concentrations versus intracellular BCG number/well (C, E) or BCG^+ cell number/well (D, F) were plotted. A non-linear curve was fitted to the dose-response data of intracellular BCG number/well at day 7 by a four-parameter logistic equation and the IC_{50} calculated (E).

Figure S1

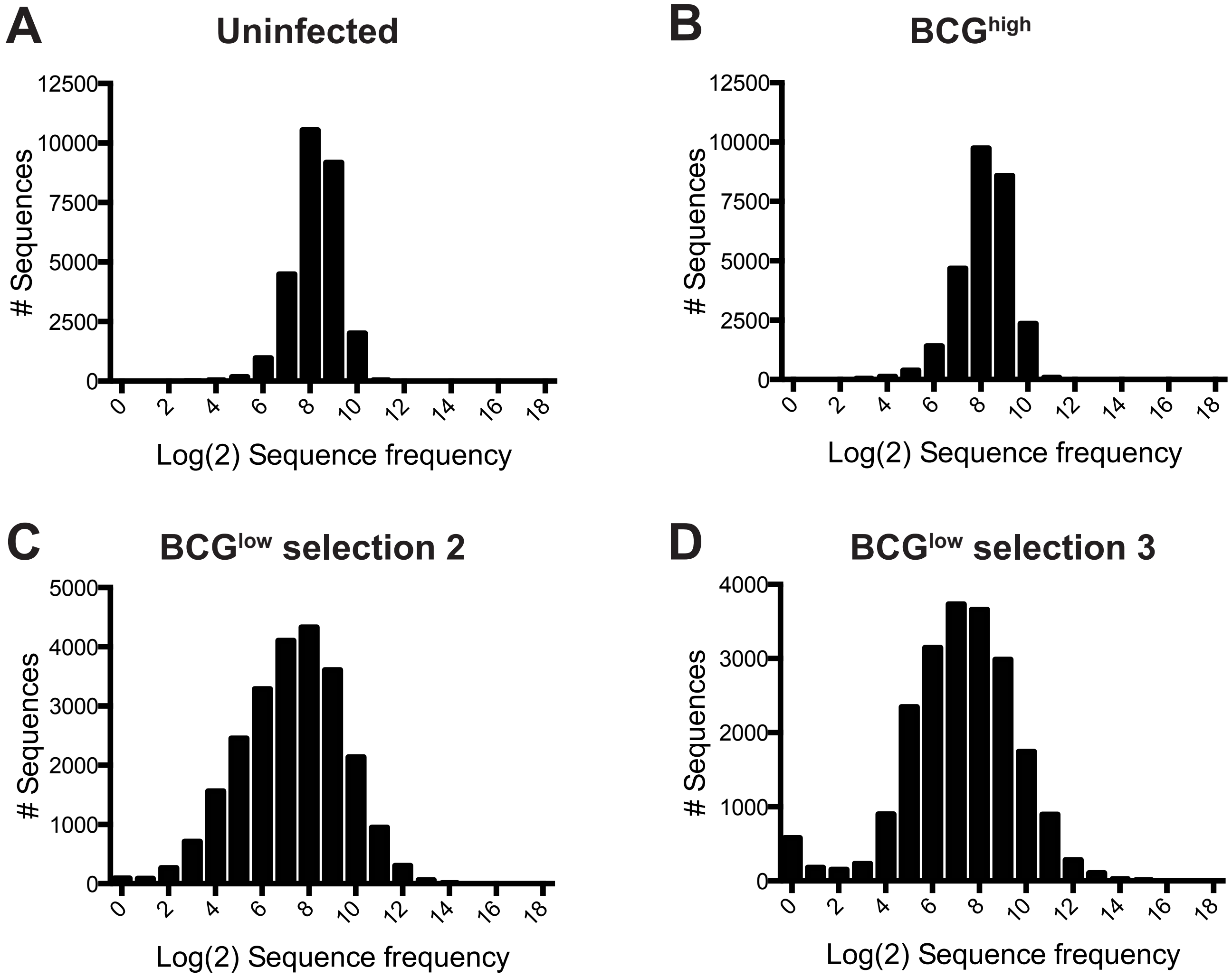
A
Uninfected
B
BCG^high
C
BCG^low selection 2
D
BCG^low selection 3
Sequences
Log(2) Sequence frequency
12500
10000
7500
5000
2500
4000
3000
2000
1000
0
2
4
6
8
10
12
14
16
18

Figure S2

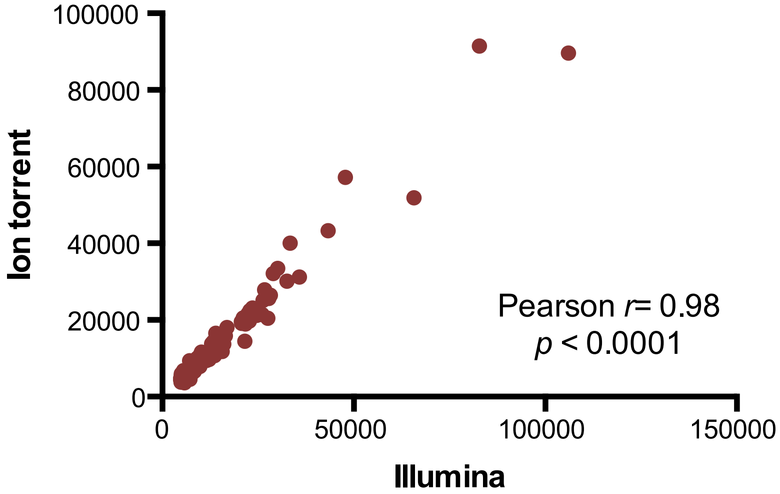

100000
80000
60000
40000
20000
0
Ion torrent
0
50000
100000
150000
Illumina
Pearson r= 0.98
p < 0.0001

Figure S3

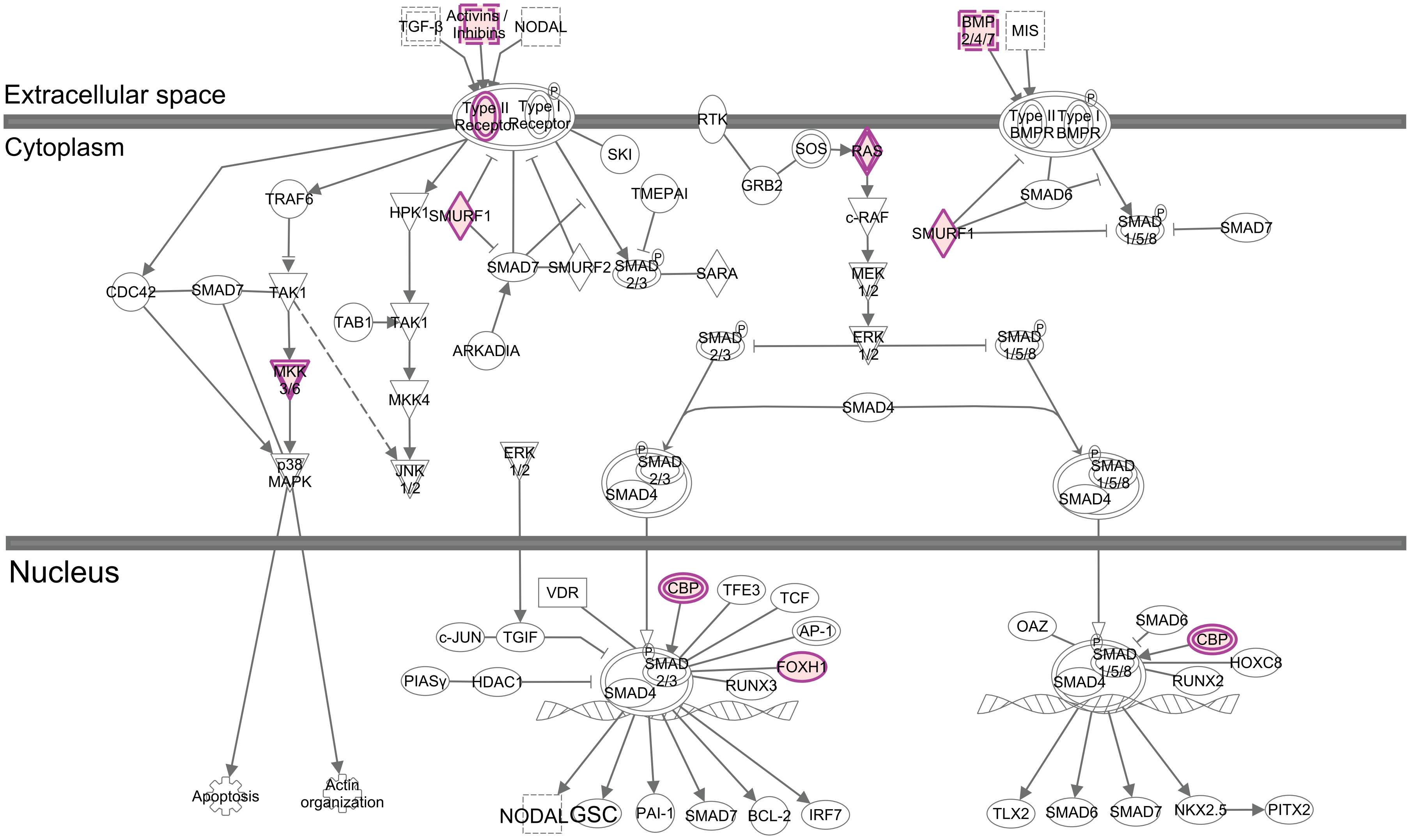

Extracellular space
Cytoplasm
Nucleus
TGF-β
Activins / Inhibins
NODAL
BMP 2/4/7
MIS
Type II Receptor
Type I Receptor
Type II BMPR
Type I BMPR
RTK
SKI
GRB2
SOS
RAS
TRAF6
HPK1
SMURF1
TMEPAI
c-RAF
SMAD6
SMAD 1/5/8
SMAD7
CDC42
TAK1
SMURF2
SMAD 2/3
SARA
MEK 1/2
TAB1
ARKADIA
ERK 1/2
MKK 3/6
MKK4
SMAD4
p38 MAPK
JNK 1/2
VDR
CBP
TFE3
TCF
AP-1
FOXH1
RUNX3
c-JUN
TGIF
PIASγ
HDAC1
OAZ
HOXC8
RUNX2
Apoptosis
Actin organization
GSC
PAI-1
BCL-2
IRF7
TLX2
NKX2.5
PITX2

Figure S4

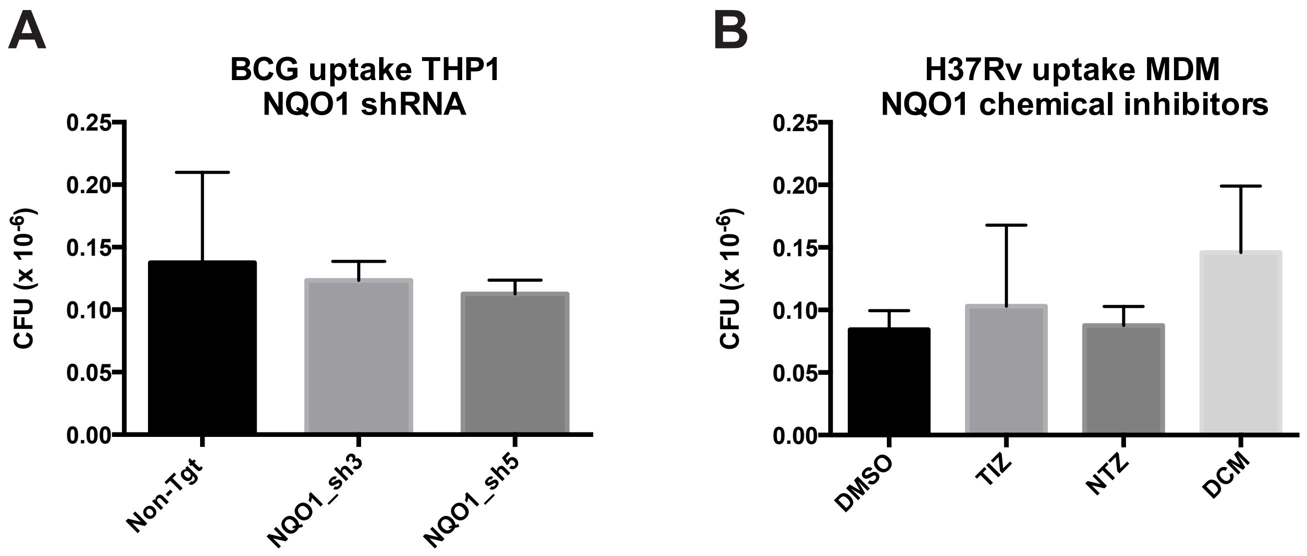
A
BCG uptake THP1
NQO1 shRNA
CFU (x 10-6)
0.25
0.20
0.15
0.10
0.05
0.00
Non-Tgt
NQO1_sh3
NQO1_sh5
B
H37Rv uptake MDM
NQO1 chemical inhibitors
CFU (x 10-6)
0.25
0.20
0.15
0.10
0.05
0.00
DMSO
TIZ
NTZ
DCM

Figure S5

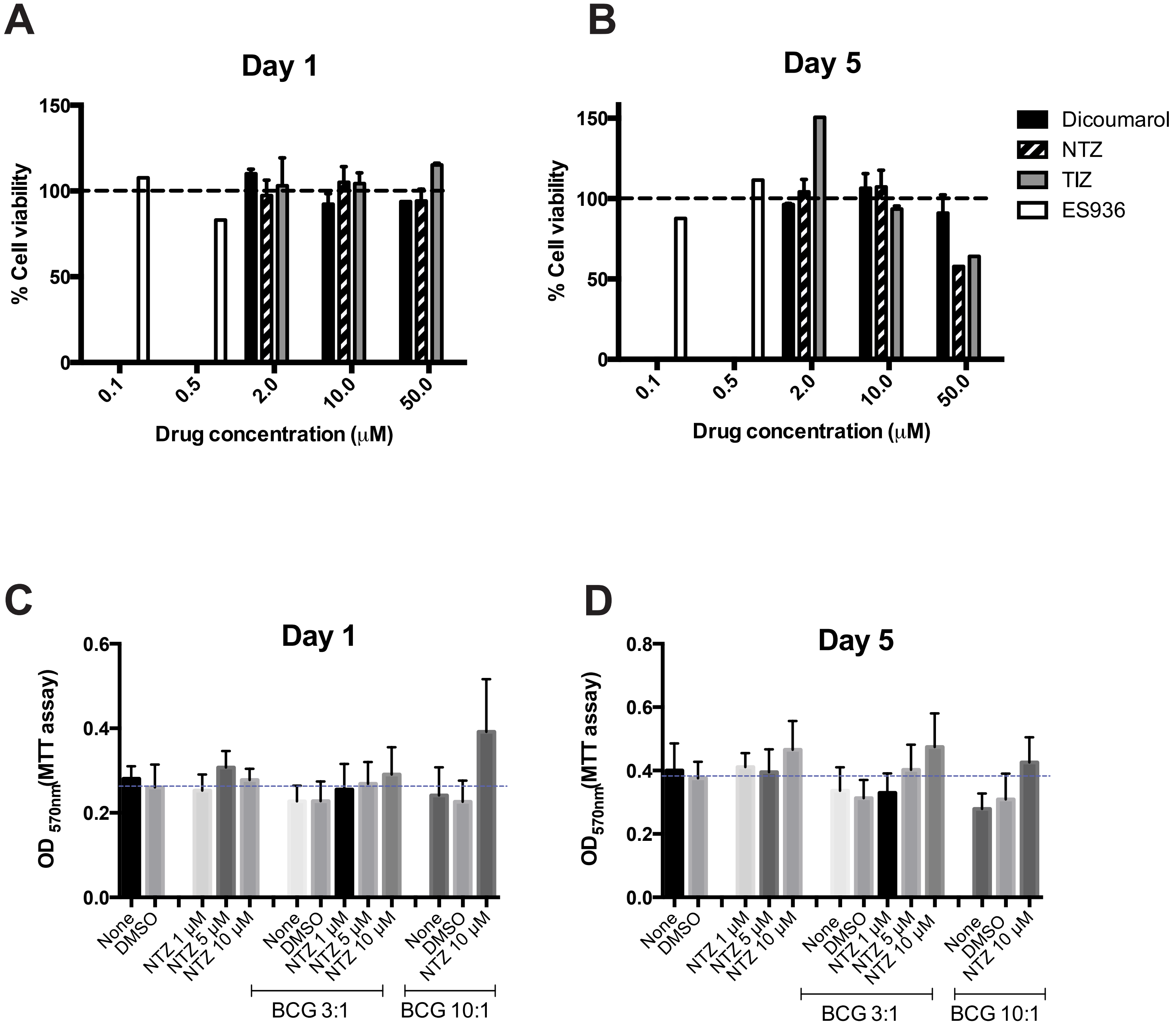
A
Day 1
150
100
50
0
% Cell viability
0.1
0.5
2.0
10.0
50.0
Drug concentration (μM)
B
Day 5
150
100
50
0
% Cell viability
0.1
0.5
2.0
10.0
50.0
Drug concentration (μM)
Dicoumarol
NTZ
TIZ
ES936
C
Day 1
0.6
0.4
0.2
0.0
OD 570nm(MTT assay)
None
DMSO
NTZ 1 μM
NTZ 5 μM
NTZ 10 μM
None
DMSO
NTZ 1 μM
NTZ 5 μM
NTZ 10 μM
None
DMSO
NTZ 10 μM
BCG 3:1
BCG 10:1
D
Day 5
0.8
0.6
0.4
0.2
0.0
OD570nm(MTT assay)
None
DMSO
NTZ 1 μM
NTZ 5 μM
NTZ 10 μM
None
DMSO
NTZ 1 μM
NTZ 5 μM
NTZ 10 μM
None
DMSO
NTZ 10 μM
BCG 3:1
BCG 10:1

Figure S6

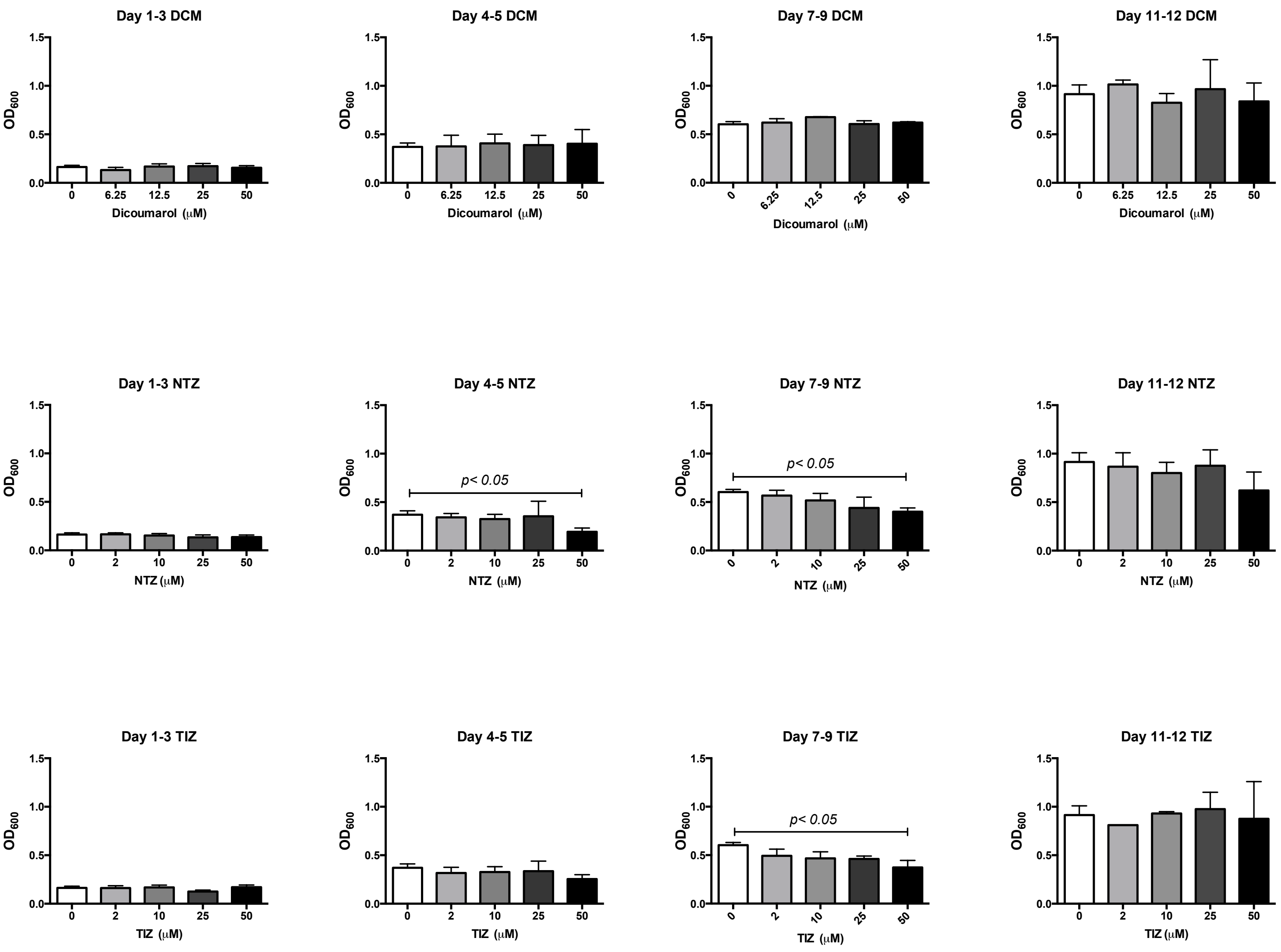
Day 1-3 DCM
Day 4-5 DCM
Day 7-9 DCM
Day 11-12 DCM
OD600
1.5
1.0
0.5
0.0
0
6.25
12.5
25
50
Dicoumarol (μM)
Day 1-3 NTZ
Day 4-5 NTZ
Day 7-9 NTZ
Day 11-12 NTZ
0
2
10
25
50
NTZ (μM)
p< 0.05
Day 1-3 TIZ
Day 4-5 TIZ
Day 7-9 TIZ
Day 11-12 TIZ
TIZ (μM)

Figure S7

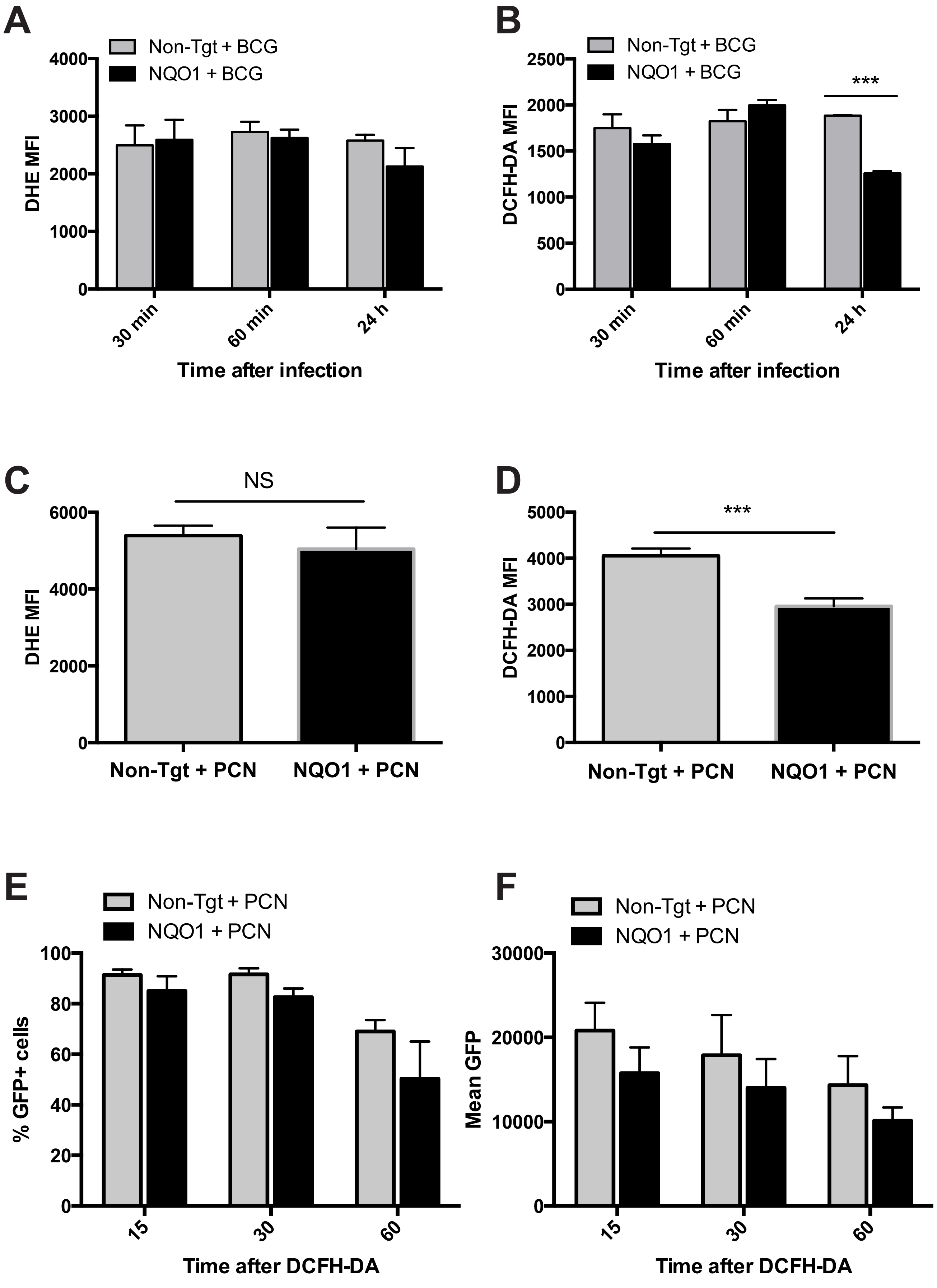
A
Non-Tgt + BCG
NQO1 + BCG
DHE MFI
4000
3000
2000
1000
0
30 min
60 min
24 h
Time after infection
B
Non-Tgt + BCG
NQO1 + BCG
DCFH-DA MFI
2500
2000
1500
1000
500
0
***
30 min
60 min
24 h
Time after infection
C
NS
DHE MFI
6000
4000
2000
0
Non-Tgt + PCN
NQO1 + PCN
D
***
DCFH-DA MFI
5000
4000
3000
2000
1000
0
Non-Tgt + PCN
NQO1 + PCN
E
Non-Tgt + PCN
NQO1 + PCN
% GFP+ cells
100
80
60
40
20
0
15
30
60
Time after DCFH-DA
F
Non-Tgt + PCN
NQO1 + PCN
Mean GFP
30000
20000
10000
0
15
30
60
Time after DCFH-DA

Figure S8

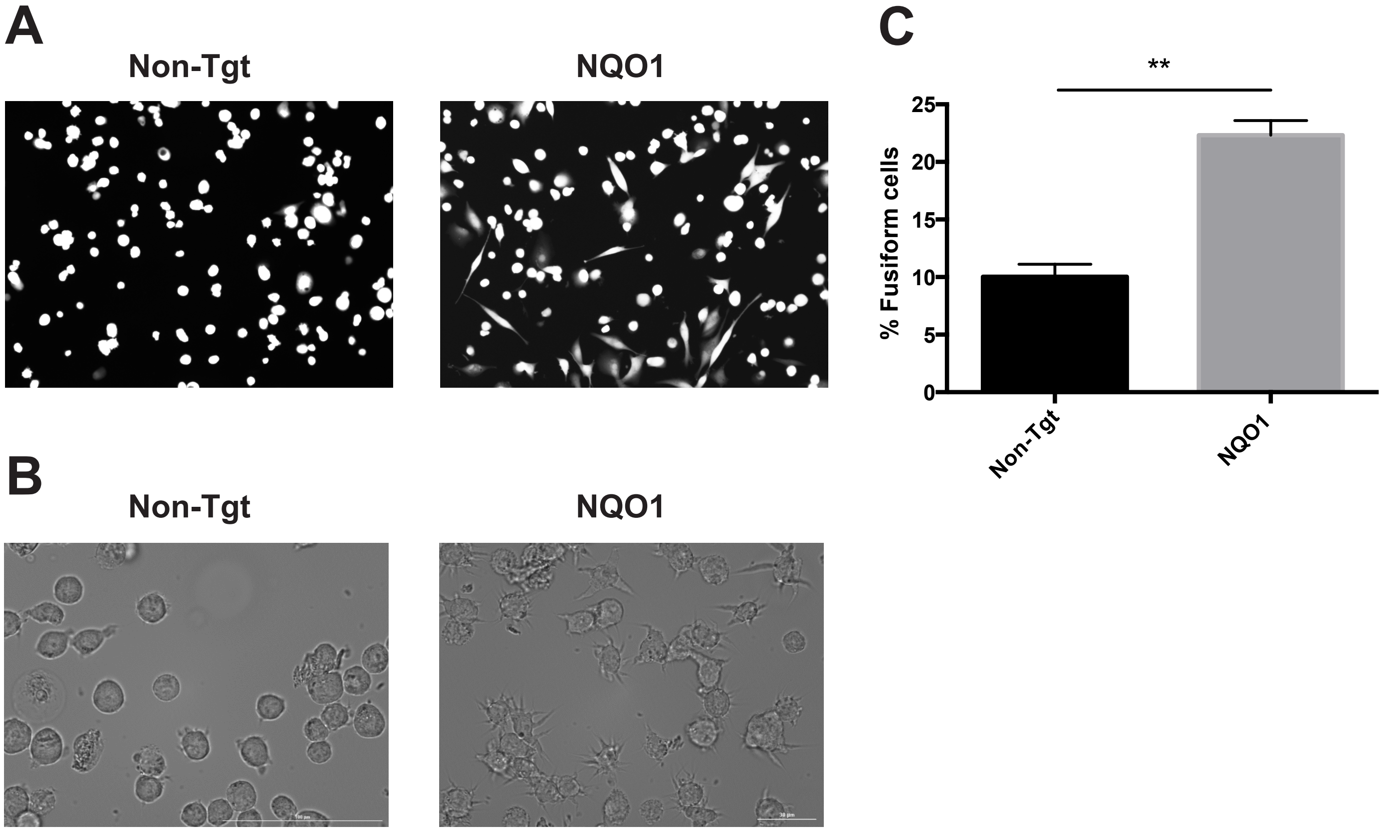
A
Non-Tgt
NQO1
B
Non-Tgt
NQO1
C
**
% Fusiform cells
0
5
10
15
20
25
Non-Tgt
NQO1

Figure S9

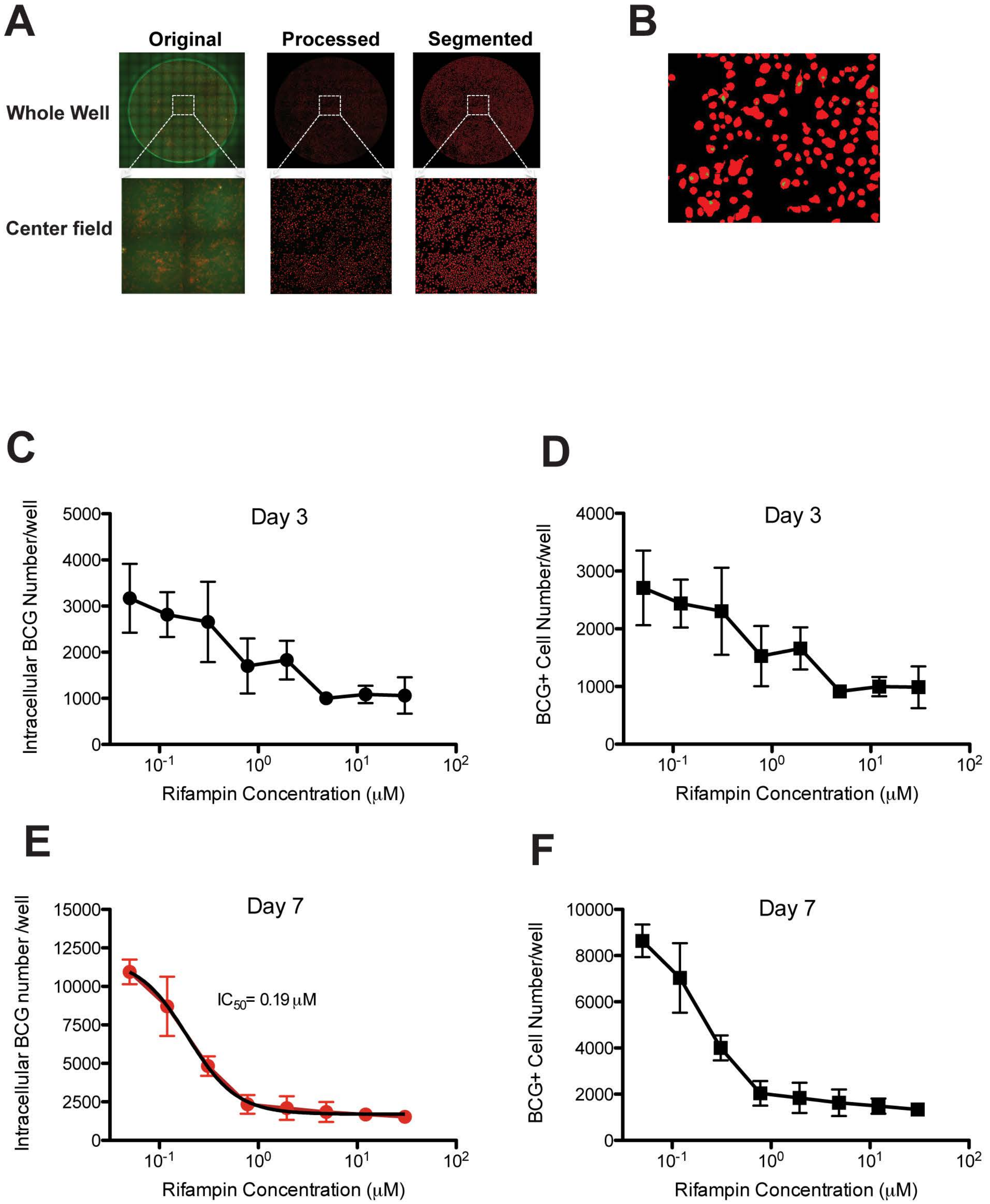
A
Original
Processed
Segmented
Whole Well
Center field
B
C
Day 3
Intracellular BCG Number/well
Rifampin Concentration (μM)
D
Day 3
BCG+ Cell Number/well
Rifampin Concentration (μM)
E
Day 7
Intracellular BCG number /well
IC50= 0.19 μM
Rifampin Concentration (μM)
F
Day 7
BCG+ Cell Number/well
Rifampin Concentration (μM)

Table S2. Sequence yields and library coverage: comparison between Illumina and Ion Torrent sequencing

Condition	Total # barcode sequences Illumina (x 10^{-6})	Total # barcode sequences IT	# different shRNAs Illumina	# different shRNAs IT	Sequence frequency range Illumina	Sequence frequency range IT
Uninfected	35.02	488,369	27,495	27,410	6-8,281	1-110
Sort 1 BCG^{High}	29.9	303,493	27,483	27,005	1-8,046	1-90
Sort 2 BCG^{Low}	17.61	266,886	24,502	20,948	1-107,809	1-1,405
Sort 3 BCG^{Low}	25.3	420,844	22,666	19,673	1-268,377	1-3,850
